# Intensive Longitudinal Methods Among Adults With Breast or Lung Cancer: Scoping Review

**DOI:** 10.2196/50224

**Published:** 2024-06-12

**Authors:** Joran Geeraerts, Kim de Nooijer, Lara Pivodic, Mark De Ridder, Lieve Van den Block

**Affiliations:** 1 End-of-Life Care Research Group Vrije Universiteit Brussel Brussels Belgium; 2 Department of Radiotherapy Universitair Ziekenhuis Brussel, Vrije Universiteit Brussel Brussels Belgium

**Keywords:** diary, ecological momentary assessment, neoplasms, quality of life, self-report, telemedicine, scoping review, longitudinal methods, breast cancer, lung cancer, patients with cancer, cancer, intensive monitoring, advanced disease stages, mobile phone

## Abstract

**Background:**

Intensive longitudinal methods offer a powerful tool for capturing daily experiences of individuals. However, its feasibility, effectiveness, and optimal methodological approaches for studying or monitoring experiences of oncology patients remain uncertain.

**Objective:**

This scoping review aims to describe to what extent intensive longitudinal methods with daily electronic assessments have been used among patients with breast or lung cancer and with which methodologies, associated outcomes, and influencing factors.

**Methods:**

We searched the electronic databases (PubMed, Embase, and PsycINFO) up to January 2024 and included studies reporting on the use of these methods among adults with breast or lung cancer. Data were extracted on population characteristics, intensive monitoring methodologies used, study findings, and factors influencing the implementation of these methods in research and clinical practice.

**Results:**

We identified 1311 articles and included 52 articles reporting on 41 studies. Study aims and intensive monitoring methodologies varied widely, but most studies focused on measuring physical and psychological symptom constructs, such as pain, anxiety, or depression. Compliance and attrition rates seemed acceptable for most studies, although complete methodological reporting was often lacking. Few studies specifically examined these methods among patients with advanced cancer. Factors influencing implementation were linked to both patient (eg, confidence with intensive monitoring system) and methodology (eg, option to use personal devices).

**Conclusions:**

Intensive longitudinal methods with daily electronic assessments hold promise to provide unique insights into the daily lives of patients with cancer. Intensive longitudinal methods may be feasible among people with breast or lung cancer. Our findings encourage further research to determine optimal conditions for intensive monitoring, specifically in more advanced disease stages.

## Introduction

### Background

People diagnosed with cancer, among which breast and lung cancer are the most prevalent diagnoses globally [1], often experience various problems and concerns that affect their quality of life and well-being across physical, psychological, social, and spiritual domains [2-6]. Understanding the fluctuations, interactions, and contextual variations of the multidimensional problems and concerns in patients’ daily lives is crucial to gain a comprehensive view of these patients’ quality of life and to optimize patient-centered care. Such insights could lead to, among others, improvements in drug schedules and personalized treatment decision-making [7] and the identification of novel care intervention targets by identifying contexts or states that aggravate or buffer against certain problems and concerns [8].

An effective way to gather insights into the daily and within-day variability of patients’ quality of life and well-being is the use of intensive longitudinal methods. Bolger and Laurenceau [9] defined intensive longitudinal methods as “an umbrella term to encompass data collection methods that employ enough repeated measurements to model a change process for each subject.” The authors specify a minimum number of 5 sequential assessments, as it enables the estimation of linear models within each participant [9]. Examples of such methods are daily diaries and ecological momentary assessments (EMAs), also known as experience sampling methods (ESM). While predominantly developed in psychological research, these methods recently gained more attention in other fields and clinical practice, including oncology, due to advancements in handheld computer technologies that enable easier implementation than traditional pencil-and-paper approaches [9-13]. Despite easier implementation of these methods, researchers and clinicians in the field of oncology still lack a clear understanding of available options for intensive longitudinal monitoring, their opportunities, pitfalls, and feasibility in populations experiencing high symptom burden. This underscores the need for a structured overview of the use and capabilities of these methods.

Currently, no systematically conducted literature review exists on the use of intensive longitudinal methods in monitoring people with cancer. One systematic review [14] provided the most recent overview of the use of EMA in people with cancer across 42 studies (23 and 8 studies included people with breast and lung cancer, respectively) and found considerable heterogeneity in the methodologies used. However, due to its inclusion criteria focusing solely on EMAs, a large group of studies monitoring patients on a once-daily basis was left out [14]. Furthermore, the review did not report on the barriers and facilitators that were encountered during the implementation of ESM, which is crucial information for optimal use in practice [14].

### Objective

We aimed to describe to what extent intensive longitudinal methods with daily electronic assessments have been used among patients with breast or lung cancer, along with the methodologies used, associated outcomes, and influencing factors. We limited the scope of this review to these patient groups with the most prevalent cancer diagnoses for feasibility reasons to provide a more nuanced picture for these methods among these groups and to inform our own ongoing ESM project among these patient groups [15]. More specifically, we described (1) the characteristics of the populations with breast or lung cancer among whom intensive longitudinal methods with daily electronic assessments have been used; (2) the objectives, design, and methods used; (3) the results obtained (including study findings and response-related results); and (4) the identified barriers and facilitators for implementing these methods in clinical and research practice.

## Methods

### Overview

We conducted a scoping review using a systematic search strategy to gain insight into the extent, range, and nature of current evidence on the use of intensive longitudinal methods with daily electronic assessments in people with breast or lung cancer, rather than providing evidence for a specific research question as in systematic reviews [16,17]. This manuscript adheres to the PRISMA-ScR (Preferred Reporting Items for Systematic Reviews and Meta-Analyses extension for Scoping Reviews) [18].

### Eligibility Criteria

We included articles that met the following criteria: articles that (1) performed in people diagnosed with breast or lung cancer through self-report or proxy responding; (2) included people aged ≥18 years; (3) used *active* intensive longitudinal methods, meaning the conscious reporting of experiences rather than passive data collection through wearables without conscious participant involvement [12]; (4) collected self-reports using electronic devices or allowed participants to choose between electronic and pen-and-paper self-reports, resulting in a partial sample that opted for electronic assessments; (5) applied a measurement period of >24 hours, with ≥5 planned assessments, including at least 1 assessment per day; and (6) included original full-text articles in English, Dutch, or French.

Articles were excluded if they met one or both of the following criteria: articles that (1) were conducted in people in complete cancer remission and (2) concerned reviews, meta-analyses, notes, letters to editors, conference abstracts, or study protocols.

### Search Strategy

The initial literature search was conducted on April 7, 2022, and updated on January 19, 2024, both without restrictions for its time coverage. We searched 3 databases: PubMed, Embase, and PsycINFO. We consulted a librarian of the Vrije Universiteit Brussel for the development of the search strategy. Keywords included terms related to the population (eg, *cancer*) and methodology (eg, *ecological momentary assessment* and *daily diary*). The search strategy was validated in PubMed and translated to other databases. The full search strategy is provided in Multimedia Appendix 1.

### Study Selection

**Figure 1:**
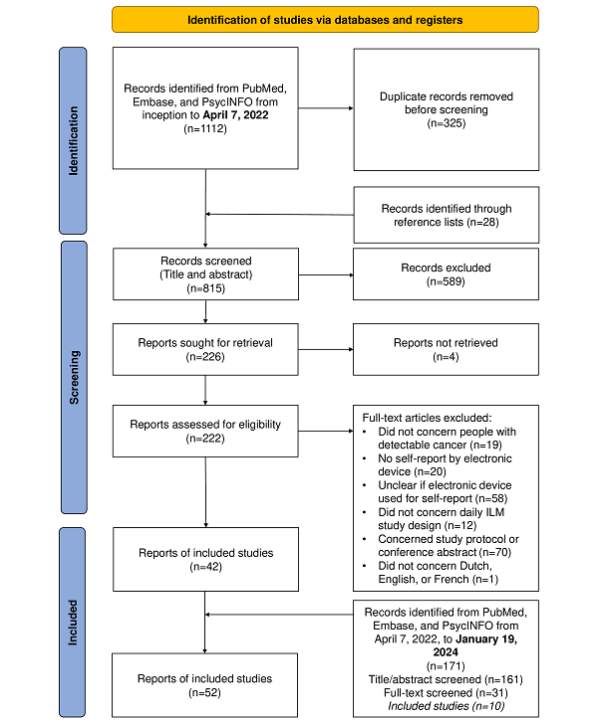
PRISMA-ScR (Preferred Reporting Items for Systematic Reviews and Meta-Analyses extension for Scoping Reviews) flow diagram. ILM: intensive longitudinal method.

Figure 1 provides an overview of the selection procedure. Most duplicates were automatically detected and removed using EndNote (version 20; Clarivate) [19]. Screening followed a 2-step process. First, 2 researchers (JG and KdN) independently screened titles and abstracts and labeled them as relevant, irrelevant, or potentially relevant for inclusion. Additional duplicates not detected by EndNote were removed during this step. Second, both reviewers screened the full texts of relevant and potentially relevant studies for final inclusion. JG and KdN resolved discrepancies in both steps through discussion and consensus and consulted a third and fourth reviewer (LP and LVdB), if necessary. JG screened articles found during the updated search. We used Rayyan (Qatar Computing Research Institute) [20] for reference management and manual removal of duplicates.

### Data Extraction and Synthesis

JG extracted data into a precreated MS Excel (version 16; Microsoft Corp) spreadsheet. To ensure consistency and accuracy of the initial search, KdN independently completed the data extraction form for a random 10% sample of included articles. JG and KdN discussed and resolved discrepancies. JG extracted updated search results. The data extraction form was revised throughout the review process. It included study characteristics (ie, authors, year, country, and overarching study); sample characteristics (ie, sample size, site of primary tumor, stage of disease, mean age, proportion of female participants, and comparison group characteristics); study aims and design; system characteristics (ie, device, application, and operation system); daily questionnaire characteristics (ie, number of items, constructs measured daily, existing measurement instruments, or sources used); sampling schedule characteristics (ie, number of monitoring periods, duration of the monitoring periods, type of sampling scheme [ie, fixed or random signal-contingent, event-contingent, or interval-contingent] [9], daily prompt frequency, and approximate time interval between prompts); supportive features for participants; response-related results (ie, participation rate, attrition rate, proportion of completed prompts, and monetary incentives); and main study findings. We listed the barriers and facilitators for the implementation of the used method in research and clinical practice per study.

We have presented the study and sample characteristics, system and sampling schedule characteristics, and response-related results in the *Results* section, grouping articles reporting on the same study. We conducted content analysis on the extracted barriers and facilitators, inductively categorizing the content in themes and subthemes.

## Results

Of the 1311 identified articles, we screened 253 (19.3%) full-text articles for eligibility. We included 52 articles, describing 41 unique studies (Figure 1).

### Population Characteristics

All the 41 studies were conducted in high-income countries, except for 1 (2%) study in Türkiye [21] (all study and sample characteristics are listed in Multimedia Appendix 2) [21-72]. We included 21 (51%) studies [22-44] reporting on samples of people with mixed primary tumor sites (including breast and lung cancer), 16 (39%) studies [21,45-66] on samples of people with breast cancer only, and 4 (10%) studies [67-72] on a sample of lung cancer only (Table 1). A total of 7 (17%) studies included patients’ partners [25,38,39,43,45-50,63]. While 26 (63%) studies were conducted in people at differing stages of disease, of which 11 (42%) included up to stage III [21,48-51,54-59,61,63,73] and 15 (58%) included up to stage IV [23,25,26,31,32,34,36-39,41-44,53,64,69,70], 6 (15%) studies [24,30,33,35,45-47,52] specifically focused on people with stage IV cancer. Sample sizes ranged from 4 [29] to 344 [42] participants, with a mean of 54.3 (SD 56.4). The mean ages were 51 (SD 4.7) years for patients with breast cancer, 65 (SD 2.8) years for patients with lung cancer, and 58 (SD 5.7) years for patients with mixed primary tumor sites. None of the studies used proxy responding.

**Table 1:** Study and sample characteristics of the included studies (n=41).

Characteristics			Frequency of studies, n (%)		Reference, year
**Primary tumor sites**					
	Breast	16 (39)		Badr et al [45], 2010; Badr et al [46], 2013; Stephenson et al [47], 2018 Belcher et al [48], 2011; Pasipanodya et al [49], 2012 Otto et al [50], 2015 Cai et al [51], 2020 Carson et al [52], 2021 Çınar et al [21], 2021 Dasch et al [53], 2010 Kim et al [60], 2016 Lim et al [64], 2022 Min et al [61], 2014 Pinto et al [54], 2021 Ratcliff et al [55], 2014 Solk et al [56], 2019; Phillips et al [57], 2020; Auster-Gussman et al [58], 2022; Welch et al [65], 2023; Whitaker et al [66], 2023 Stone et al [59], 2016 Sztachańska et al [62], 2019 Xu et al [63], 2019	
	Lung	4 (10)		Dunsmore et al [72], 2023 Maguire et al [71], 2015 Shiyko et al [68], 2014; Shiyko et al [67], 2019 Steffen et al [69], 2018; Steffen et al [70], 2020	
	Mixed	21 (51)		Aigner et al [22], 2016 Besse et al [34], 2016 Chumbler et al [23], 2007 Coolbrandt et al [40], 2022 Hachizuka et al [35], 2010 Harper et al [24], 2012 Kearney et al [28], 2006 Langer et al [25], 2018 LeBaron et al [38], 2022; LeBaron et al [39], 2023 Lee et al [41], 2023 Maguire et al [29], 2005 McCall et al [30], 2008 McCann et al [31], 2009; Kearney et al [32], 2009 Mooney et al [26], 2014 Nordhausen et al [42], 2022 Passardi et al [36], 2022 Schuler et al [43], 2023 van den Berg et al [27], 2022 van Roozendaal et al [44], 2023 Weaver et al [33], 2014 Yap et al [37], 2013	
Included patients and partners			7 (17)		Badr et al [45], 2010; Badr et al [46], 2013; Stephenson et al [47], 2018; Belcher et al [48], 2011; Pasipanodya et al [49], 2012 Otto et al [50], 2015 Langer et al [25], 2018; LeBaron et al [38], 2022 LeBaron et al [39], 2023 Schuler et al [43], 2023 Xu et al [63], 2019
**Disease stage**					
	I to II	1 (2)		Shiyko et al [68], 2014; Shiyko et al [67], 2019	
	III to IV	9 (22)		Badr et al [45], 2010; Badr et al [46], 2013; Stephenson et al [47], 2018 Carson et al [52], 2021 Hachizuka et al [35], 2010 Harper et al [24], 2012 LeBaron et al [38], 2022; LeBaron et al [39], 2023 Lim et al [64], 2022 McCall et al [30], 2008 Schuler et al [43], 2023 Weaver et al [33], 2014	
	Mixed	20 (49)		Belcher et al [48], 2011; Pasipanodya et al [49], 2012 Otto et al [50], 2015 Besse et al [34], 2016 Cai et al [51], 2020 Chumbler et al [23], 2007 Çınar et al [21], 2021 Coolbrandt et al [40], 2022 Dasch et al [53], 2010 Langer et al [25], 2018 McCann et al [31], 2009; Kearney et al [32], 2009 Min et al [61], 2014 Mooney et al [26], 2014 Pinto et al [54], 2021 Ratcliff et al [55], 2014 Solk et al [56], 2019; Phillips et al [57], 2020; Auster-Gussman et al [58], 2022; Welch et al [65], 2023; Whitaker et al [63], 2023 Steffen et al [69], 2018; Steffen et al [70], 2020 Stone et al [59], 2016 van Roozendaal et al [44], 2023 Xu et al [63], 2019 Yap et al [37], 2013	
	Not fully mentioned	11 (27)		Aigner et al [22], 2016 Dunsmore et al [72], 2023 Kearney et al [28], 2006 Kim et al [60], 2016 Lee et al [41], 2023 Maguire et al [29], 2005 Maguire et al [71], 2015 Nordhausen et al [42], 2022 Passardi et al [36], 2022 Sztachańska et al [62], 2019 van den Berg et al [27], 2022	
**Sample size**					
	4-20	9 (22)		Besse et al [34], 2016 Cai et al [51], 2020 Hachizuka et al [35], 2010 Kearney et al [28], 2006 LeBaron et al [38], 2022; LeBaron et al [39], 2023 Maguire et al [29], 2005 Maguire et al [71], 2015 Ratcliff et al [55], 2014 Schuler et al [43], 2023	
	21-50	14 (34)		Aigner et al [22], 2016 Carson et al [52], 2021 Chumbler et al [23], 2007 Çınar et al [21], 2021 Dunsmore et al [72], 2023 Harper et al [24], 2012 McCall et al [30], 2008 Min et al [61], 2014 Passardi et al [36], 2022 Pinto et al [54], 2021 Steffen et al [69], 2018; Steffen et al [70], 2020 Sztachańska et al [62], 2019 van Roozendaal et al [44], 2023 Weaver et al [33], 2014	
	51-100	13 (32)		Badr et al [45], 2010; Badr et al [46], 2013; Stephenson et al [47], 2018 Belcher et al [48], 2011; Pasipanodya et al [49], 2012 Otto et al [50], 2015 Dasch et al [53], 2010 Kim et al [60], 2016 Lim et al [64], 2022 McCann et al [31], 2009; Kearney et al [32], 2009 Shiyko et al [68], 2014; Shiyko et al [67], 2019 Solk et al [56], 2020; Phillips et al [57], 2020; Auster-Gussman et al [58], 2022; Welch et al [65], 2023; Whitaker et al [66], 2023 Stone et al [59], 2016 van den Berg et al [27], 2022 Xu et al [63], 2019 Yap et al [37], 2013	
	100-344	5 (12)		Coolbrandt et al [40], 2022 Langer et al [25], 2018 Lee et al [41], 2023 Mooney et al [26], 2014 Nordhausen et al [42], 2022	
**Mean age (years)**					
	40-50	10 (24)		Badr et al [45], 2010; Badr et al [46], 2013; Stephenson et al [47], 2018 Çınar et al [21], 2021 Kim et al [60], 2016 Langer et al [25], 2018 Min et al [61], 2014 Solk et al [56], 2019; Phillips et al [57], 2020; Auster-Gussman et al [58], 2022; Welch et al [65], 2023; Whitaker et al [66], 2023 Sztachańska et al [62], 2019 van Roozendaal et al [44], 2023 Xu et al [63], 2019 Yap et al [37], 2013	
	51-60	19 (46)		Aigner et al [22], 2016 Belcher et al [48], 2011; Pasipanodya et al [49], 2012 Otto et al [50], 2015 Besse et al [34], 2016 Cai et al [51], 2020 Carson et al [52], 2021 Coolbrandt et al [40], 2022 Dasch et al [53], 2010 Harper et al [24], 2012 Lee et al [41], 2023 Lim et al [64], 2022 McCann et al [31], 2009; Kearney et al [32], 2009 Mooney et al [26], 2014 Pinto et al [54], 2021 Ratcliff et al [55], 2014 Schuler et al [43], 2023 Stone et al [59], 2016 van den Berg et al [27], 2022 Weaver et al [33], 2014	
	61-70	9 (22)		Chumbler et al [23], 2007 Dunsmore et al [72], 2023 Hachizuka et al [35], 2010 Maguire et al [71], 2015 McCall et al [30], 2008 Nordhausen et al [42], 2022 Passardi et al [36], 2022 Shiyko et al [68], 2014; Shiyko et al [67], 2019 Steffen et al [69], 2018; Steffen et al [70], 2020	
	Not mentioned	3 (7)		Kearney et al [28], 2006 LeBaron et al [38], 2022; LeBaron et al [39], 2023 Maguire et al [29], 2005	
**Study design as reported by study authors**					
	Observational	30 (73)		Aigner et al [22], 2016 Badr et al [45], 2010; Badr et al [46], 2013; Stephenson et al [47], 2018 Belcher et al [48], 2011; Pasipanodya et al [49], 2012 Otto et al [50], 2015 Chumbler et al [23], 2007 Coolbrandt et al [40], 2022 Dasch et al [53], 2010 Dunsmore et al [72], 2023 Hachizuka et al [35], 2010 Harper et al [24], 2012 Kearney et al [28], 2006 Kim et al [60], 2016 Langer et al [25], 2018 LeBaron et al [38], 2022; LeBaron et al [39], 2023 Lee et al [41], 2023 Maguire et al [71], 2015 McCall et al [30], 2008 Min et al [61], 2014 Nordhausen et al [42], 2022 Pinto et al [54], 2021 Ratcliff et al [55], 2014 Schuler et al [43], 2023 Shiyko et al [68], 2014; Shiyko et al [67], 2019 Solk et al [56], 2019; Phillips et al [57], 2020; Auster-Gussman et al [58], 2022; Welch et al [65], 2023; Whitaker et al [66], 2023 Steffen et al [69], 2018; Steffen et al [70], 2020 Stone et al [59], 2016 van den Berg et al [27], 2022 van Roozendaal et al [44], 2023 Xu et al [63], 2019 Yap et al [37], 2013	
	Interventional	11 (27)		Besse et al [34], 2016 Cai et al [51], 2020 Carson et al [52], 2021 Çınar et al [21], 2021 Lim et al [64], 2022 Maguire et al [29], 2005 McCann et al [31], 2009; Kearney et al [32], 2009 Mooney et al [26], 2014 Passardi et al [36], 2022 Sztachańska et al [62], 2019 Weaver et al [33], 2014	
**Study objectives**					
	Feasibility, usability, or validity	19 (46)		Besse et al [34], 2016 Cai et al [51], 2020 Chumbler et al [23], 2007 Coolbrandt et al [40], 2022 Hachizuka et al [35], 2010 Harper et al [24], 2012 Kearney et al [28], 2006 Kim et al [60], 2016 LeBaron et al [38], 2022 Lee et al [41], 2023 Maguire et al [29], 2005 McCall et al [30], 2008 Min et al [61], 2014 Nordhausen et al [42], 2022 Passardi et al [36], 2022 Schuler et al [43], 2023 Solk et al [56], 2019 Stone et al [59], 2016 van den Berg et al [27], 2022 Yap et al [37], 2013	
	Trajectory or relationship of variables	17 (41)		Aigner et al [22], 2016 Badr et al [45], 2010; Badr et al [46], 2013; Stephenson et al [47], 2018 Belcher et al [48], 2011; Pasipanodya et al [49], 2012 Otto et al [50], 2015 Dasch et al [53], 2010 Dunsmore et al [72], 2023 Langer et al [25], 2018 LeBaron et al [39], 2023 Pinto et al [54], 2021 Ratcliff et al [55], 2014 Shiyko et al [67], 2019 Phillips et al [57], 2020; Auster-Gussman et al [58], 2022; Welch et al [65], 2023; Whitaker et al [66], 2023 Steffen et al [69], 2018; Steffen et al [70], 2020 van Roozendaal et al [44], 2023 Weaver et al [33], 2014 Xu et al [63], 2019	
	Effectiveness of methods as intervention	4 (10)		Çınar et al [21], 2021 McCann et al [31], 2009; Kearney et al [32], 2009 Mooney et al [26], 2014 Maguire et al [71], 2015	
	Effectiveness of other interventions	3 (7)		Carson et al [52], 2021 Lim et al [64], 2022 Sztachańska et al [62], 2019	
	Introduce statistical approach	1 (2)		Shiyko et al [68], 2014	

### Study Design and Objectives

Of the 41 studies, 30 (73%) [22-25,27,28,30,37-50,53-61,63,65-67,69-72] used intensive methods in observational study designs, whereas 11 (27%) [21,26,29,31-34,36,51,52,62,64] used them in interventional studies. While 38% (20/52) of the articles [23,24,27-30,33,34,36-38,40-43,51,56,59-61] focused on the intensive method’s feasibility, usability, or validity, other articles investigated the prevalence or trajectory of measured variables or relationships between those variables [22,25,39,44-50,53-55,57,58,63,65-67,69,70,72], the effectiveness of the intensive methods as an intervention [21,26,31,32,71], or the effectiveness of other interventions [52,62,64] or introduced a novel statistical approach [68].

### Data Collection Methods

#### Daily Measured Constructs

Of the 41 studies, 30 (73%) [23-25,27-34,36,37,40-42,44-50,52-59,62,63,65-72] used items adapted from previous studies or scales (study details are listed in Multimedia Appendix 3) [21-72]. Some of the most frequently recurring questionnaires were the Common Toxicity Criteria Adverse Events grading system [29,31-33,37,41,74], EORTC Core Quality of Life Questionnaire (EORTC-QLQ-C30) [24,42,69,70,75], and the Positive and Negative Affect Schedule-Expanded scale [48-50,53,69,70,76]. Measured constructs covered physical, psychological, and social domains; behaviors and intentions; daily events; sleep quality; and general quality of life. The physical domain was the most assessed domain, with the most frequently measured constructs being pain [22,23,26,27,30,34,35,38-41,45-47,52,54,56,57,59,65-68,70] and fatigue [23,26-32,35,40,41,44,54-57,59,65,66,70]. Anxiety [22,26,35,41,51,54,56,57,59-61,65,66,72] and depression [26,35,51,56,57,59,61,65,66] were the most frequently measured constructs in the psychological domain, and social support [25,45,48,50,62] and communication [25,38,39,46,49,50,63] were the most frequently measured constructs in the social domain. Frequently measured behavioral constructs included medication use [22,36,38-40,47,61,64] and physical activity [38,39,56,57,65,66].

#### Sampling Schedule Characteristics

Of the 41 studies, 23 (56%) [21-24,26-28,30,37-41,43,48,50-53,59,61,62,69,70,72] required patients to fill in the questionnaire once per day, while 6 (15%) studies [25,29,31-34,67,68] required 2 completions daily, and 7 (17%) studies [35,44-47,54-58,63,65,66] required 3-6 completions daily (Table 2). Moreover, 5 (12%) studies [36,42,60,64,71] did not report the specific amount.

**Table 2:** Data collection methods used in the included studies (n=41).

Data collection methods			Frequency of studies, n (%)		Reference, year
**Sampling schedule**					
	Once daily	23 (56)		Aigner et al [22], 2016 Belcher et al [48], 2011; Pasipanodya et al [49], 2012 Otto et al [50], 2015 Cai et al [51], 2020 Carson et al [52], 2021 Chumbler et al [23], 2007 Çınar et al [21], 2021 Coolbrandt et al [40], 2022 Dasch et al [53], 2010 Dunsmore et al [72], 2023 Harper et al [24], 2012 Kearney et al [28], 2006 LeBaron et al [38], 2022; LeBaron et al [39], 2023 Lee et al [41], 2023 McCall et al [30], 2008 Min et al [61], 2014 Mooney et al [26], 2014 Schuler et al [43], 2023 Steffen et al [69], 2018; Steffen et al [70], 2020 Stone et al [59], 2016 Sztachańska et al [62], 2019 van den Berg et al [27], 2022 Yap et al [37], 2013	
	Twice daily	6 (15)		Besse et al [34], 2016 Langer et al [25], 2018 Maguire et al [29], 2005 McCann et al [31], 2009; Kearney et al [32], 2009 Shiyko et al [68], 2014; Shiyko et al [67], 2019 Weaver et al [33], 2014	
	3-6 times daily	7 (17)		Badr et al [45], 2010; Badr et al [46], 2013; Stephenson et al [47], 2018 Hachizuka et al [35], 2010 Pinto et al [54], 2021 Ratcliff et al [55], 2014 Solk et al [56], 2019; Phillips et al [57], 2020; Auster-Gussman et al [58], 2022; Welch et al [65], 2023; Whitaker et al [66], 2023 van Roozendaal et al [44], 2023 Xu et al [63], 2019	
	Not mentioned	6 (15)		Kim et al [60], 2016 Lim et al [64], 2022 Maguire et al [71], 2015 Nordhausen et al [42], 2022 Passardi et al [36], 2022	
**Sampling type^a^**					
	Fixed signal-contingent	15 (37)		Belcher et al [48], 2011; Pasipanodya et al [49], 2012 Otto et al [50], 2015 Besse et al [34], 2016 Cai et al [51], 2020 Coolbrandt et al [40], 2022 Dunsmore et al [72], 2023 Hachizuka et al [35], 2010 Langer et al [25], 2018 LeBaron et al [38], 2022; LeBaron et al [39], 2023 Min et al [61], 2014 Passardi et al [36], 2022 Schuler et al [43], 2023 Steffen et al [69], 2018; Steffen et al [70], 2020 Xu et al [63], 2019 Yap et al [37], 2013	
	Random signal-contingent	7 (17)		Badr et al [45], 2010; Badr et al [46], 2013; Stephenson et al [47], 2018 Hachizuka et al [35], 2010 Pinto et al [54], 2021 Ratcliff et al [55], 2014 Shiyko et al [68], 2014; Shiyko et al [67], 2019 Solk et al [56], 2019; Phillips et al [57], 2020; Auster-Gussman et al [58], 2021; Welch et al [65], 2023; Whitaker et al [66], 2023 van Roozendaal et al [44], 2023	
	Interval-contingent	6 (15)		Çınar et al [21], 2021 Dasch et al [53], 2010 McCall et al [30], 2008 Stone et al [59], 2016 Sztachańska et al [62], 2019 Weaver et al [33], 2014	
	Event-contingent	6 (15)		Hachizuka et al [35], 2010 LeBaron et al [38], 2022; LeBaron et al [39], 2023 Maguire et al [29], 2005 McCall et al [30], 2008 McCann et al [31], 2009; Kearney et al [32], 2009 Schuler et al [43], 2023	
	Not clearly mentioned	15 (37)		Aigner et al [22], 2016 Carson et al [52], 2021 Chumbler et al [23], 2007 Harper et al [24], 2012 Kearney et al [28], 2006 Kim et al [60], 2016 Lee et al [41], 2023 Lim et al [64], 2022 Maguire et al [29], 2005 Maguire et al [71], 2015 McCann et al [31], 2009; Kearney et al [32], 2009 Mooney et al [26], 2014 Nordhausen et al [42], 2022 Passardi et al [36], 2022 van den Berg et al [27], 2022	
**Data collection period length (days)**					
	5	1 (2)		Yap et al [37], 2013	
	7	8 (20)		Belcher et al [48], 2011; Pasipanodya et al [49], 2012 Otto et al [50], 2015 Cai et al [51], 2020 Carson et al [52], 2021 Dasch et al [53], 2010 Dunsmore et al [72], 2023 Hachizuka et al [35], 2010 Kearney et al [28], 2006 Pinto et al [54], 2021	
	8-13	3 (7)		Otto et al [50], 2015 Solk et al [56], 2019; Phillips et al [57], 2020; Auster-Gussman et al [58], 2022; Welch et al [65], 2023; Whitaker et al [66], 2023 Xu et al [63], 2019	
	14	7 (17)		Aigner et al [22], 2016 Badr et al [45], 2010; Badr et al [46], 2013; Stephenson et al [47], 2018 Langer et al [25], 2018 Maguire et al [29], 2005 McCann et al [31], 2009; Kearney et al [32], 2009 Shiyko et al [68], 2014; Shiyko et al [67], 2019 Sztachańska et al [62], 2019	
	>14	12 (29)		Besse et al [34], 2016 Çınar et al [21], 2021 Lee et al [41], 2023 Lim et al [64], 2022 Maguire et al [71], 2015 McCall et al [30], 2008 Min et al [61], 2014 Schuler et al [43], 2023 Steffen et al [69], 2018; Steffen et al [70], 2020 Stone et al [59], 2016 van den Berg et al [27], 2022 Weaver et al [33], 2014	
	Variable per person	10 (24)		Chumbler et al [23], 2007 Coolbrandt et al [40], 2022 Harper et al [24], 2012 Kim et al [60], 2016 LeBaron et al [38], 2022; LeBaron et al [39], 2023 Mooney et al [26], 2014 Nordhausen et al [42], 2022 Passardi et al [36], 2022 Ratcliff et al [55], 2014 van Roozendaal et al [44], 2023	
**Data collection devices for self-report assessments**					
	Smartphone	11 (27)		Cai et al [51], 2020 Çınar et al [21], 2021 Coolbrandt et al [40], 2022 Langer et al [25], 2018 Min et al [61], 2014 Pinto et al [54], 2021 Schuler et al [43], 2023 Solk et al [56], 2019; Phillips et al [57], 2020; Auster-Gussman et al [58], 2022; Welch et al [65], 2023; Whitaker et al [66], 2023 van den Berg et al [27], 2022 van Roozendaal et al [44], 2023 Xu et al [63], 2019	
	Smartwatch	2 (5)		LeBaron et al [38], 2022; LeBaron et al [39], 2023	
	Handheld computer	8 (20)		Aigner et al [22], 2016 Badr et al [45], 2010; Badr et al [46], 2013; Stephenson et al [47], 2018 Hachizuka et al [35], 2010 Harper et al [24], 2012 Kearney et al [28], 2006 McCall et al [30], 2008 Ratcliff et al [55], 2014 Shiyko et al [68], 2014; Shiyko et al [67], 2019	
	Mobile device with telephone or SMS functionality	9 (22)		Besse et al [34], 2016 Carson et al [52], 2021 Lee et al [41], 2023 Maguire et al [29], 2005 Maguire et al [71], 2015 McCann et al [31], 2009; Kearney et al [32], 2009 Mooney et al [26], 2014 Weaver et al [33], 2014 Yap et al [37], 2013	
	Device with internet functionality	5 (12)		Belcher et al [48], 2011; Pasipanodya et al [49], 2012 Otto et al [50], 2015 Dasch et al [53], 2010 Dunsmore et al [72], 2023 Steffen et al [69], 2018; Steffen et al [70], 2020 Stone et al [59], 2016	
	Specifically developed device	2 (5)		Chumbler et al [23], 2007 Nordhausen et al [42], 2022	
	Not mentioned	5 (12)		Otto et al [50], 2015 Kim et al [60], 2016 Lim et al [64], 2022 Passardi et al [36], 2022 Sztachańska et al [62], 2019	
**Device ownership**					
	Patient-owned	19 (46)		Belcher et al [48], 2011; Pasipanodya et al [49], 2012 Otto et al [50], 2015 Besse et al [34], 2016 Cai et al [51], 2020 Carson et al [52], 2021 Çınar et al [21], 2021 Coolbrandt et al [40], 2022 Dasch et al [53], 2010 Lee et al [41], 2023 Min et al [61], 2014 Mooney et al [26], 2014 Pinto et al [54], 2021 Schuler et al [43], 2023 Solk et al [56], 2019; Phillips et al [57], 2020; Auster-Gussman et al [58], 2021; Welch et al [65], 2023; Whitaker et al [66], 2023 Stone et al [59], 2016 van den Berg et al [27], 2022 van Roozendaal et al [44], 2023 Xu et al [63], 2019 Yap et al [37], 2013	
	Provided by researcher	12 (29)		Aigner et al [22], 2016 Badr et al [45], 2010; Badr et al [46], 2013; Stephenson et al [47], 2018 Chumbler et al [23], 2007 Hachizuka et al [35], 2010 Harper et al [24], 2012 Kearney et al [28], 2006 LeBaron et al [38], 2022; LeBaron et al [39], 2023 Nordhausen et al [42], 2022 Ratcliff et al [55], 2014 Shiyko et al [68], 2014; Shiyko et al [67], 2019 Weaver et al [33], 2014	
	Option to choose between patient-owned and research device	2 (5)		Langer et al [25], 2018 Steffen et al [69], 2019; Steffen et al [70], 2020	
	Not mentioned	10 (24)		Otto et al [50], 2015 Dunsmore et al [72], 2023 Kim et al [60], 2016 Lim et al [64], 2022 Maguire et al [29], 2005 Maguire et al [71], 2015 McCall et al [30], 2008 McCann et al [31], 2009; Kearney et al [32], 2009 Passardi et al [36], 2022 Sztachańska et al [62], 2019	
**Data collection software^a^**					
	Smartphone apps	9 (22)		Çınar et al [21], 2021 Coolbrandt et al [40], 2022 Kim et al [60], 2016 Langer et al [25], 2018 Min et al [61], 2014 Pinto et al [54], 2021 Schuler et al [43], 2023 van den Berg et al [27], 2022 van Roozendaal et al [44], 2023	
	Browser-based surveys (sent via chat, mail, or SMS)	6 (15)		Belcher et al [48], 2011; Pasipanodya et al [49], 2012 Otto et al [50], 2015 Dasch et al [53], 2010 Dunsmore et al [72], 2023 Solk et al [56], 2019; Phillips et al [57], 2020; Auster-Gussman et al [58], 2022; Welch et al [65], 2023; Whitaker et al [66], 2023 Steffen et al [69], 2018; Steffen et al [70], 2020 Xu et al [63], 2019	
	SMS	3 (7)		Besse et al [34], 2016 Cai et al [51], 2020 Yap et al [37], 2013	
	Interactive voice responding systems	4 (10)		Besse et al [34], 2016 Carson et al [52], 2021 Lee et al [41], 2023 Mooney et al [26], 2014	
	Other specifically developed software	12 (29)		Aigner et al [22], 2016 Chumbler et al [23], 2007 Kearney et al [28, 2006 LeBaron et al [38], 2022; LeBaron et al [39], 2023 Maguire et al [29], 2005 Maguire et al [71], 2015 McCann et al [31], 2009; Kearney et al [32], 2009 Nordhausen et al [42], 2022 Passardi et al [36], 2022 Ratcliff et al [55], 2014 Stone et al [59], 2016 Weaver et al [33], 2014	
	Not mentioned	8 (20)		Badr et al [45], 2010; Badr et al [46], 2013; Stephenson et al [47], 2018 Otto et al [50], 2015 Hachizuka et al [35], 2010 Harper et al [24], 2012 Lim et al [64], 2022 McCall et al [30], 2008 Shiyko et al [68], 2014; Shiyko et al [67], 2019 Sztachańska et al [62], 2019	
Used conditional questionnaire items			7 (17)		Badr et al [45], 2010; Badr et al [46], 2013 Stephenson et al [47], 2018 Belcher et al [48], 2011; Pasipanodya et al [49], 2012 Otto et al [50], 2015 Coolbrandt et al [40], 2022 Langer et al [25], 2018 Mooney et al [26], 2014 Shiyko et al [68], 2014; Shiyko et al [67], 2019
Used different questionnaire lengths depending on prompt timing			5 (12)		Badr et al [45], 2010; Badr et al [46], 2013; Stephenson et al [47], 2018 Langer et al [25], 2018 Ratcliff et al [55], 2014 Schuler et al [43], 2023 Solk et al [56], 2019; Phillips et al [57], 2020; Auster-Gussman et al [58], 2022; Welch et al [65], 2023; Whitaker et al [66], 2023
**The number of questionnaire items**					
	1-20	20 (49)		Aigner et al [22], 2016 Badr et al [45], 2010; Badr et al [46], 2013; Stephenson et al [47], 2018 Besse et al [34], 2016 Carson et al [52], 2021 Hachizuka et al [35], 2010 Harper et al [24], 2012 Kim et al [60], 2016 Langer et al [25], 2018 LeBaron et al [38], 2022; LeBaron et al [39], 2023 Min et al [61], 2014 Mooney et al [26], 2014 Nordhausen et al [42], 2022 Ratcliff et al [55], 2014 Schuler et al [43], 2023 Shiyko et al [68], 2014; Shiyko et al [67], 2019 Solk et al [56], 2019; Phillips et al [57], 2020; Auster-Gussman et al [58], 2022; Welch et al [65], 2023; Whitaker et al [66], 2023 Stone et al [59], 2016 van den Berg et al [27], 2022 van Roozendaal et al [44], 2023 Yap et al [37], 2013	
	21-40	6 (15)		Dasch et al [53], 2010 Dunsmore et al [72], 2023 Lee et al [41], 2023 Pinto et al [54], 2021 Steffen et al [69], 2018; Steffen et al [70], 2020 Sztachańska et al [62], 2019	
	41-84	2 (5)		Belcher et al [48], 2011; Pasipanodya et al [49], 2012 Otto et al [50], 2015	
	Not clearly mentioned	13 (32)		Cai et al [51], 2020 Chumbler et al [23], 2007 Çınar et al [21], 2021 Coolbrandt et al [40], 2022 Kearney et al [28], 2006 Lim et al [64], 2022 Maguire et al [29], 2005 Maguire et al [71], 2015 McCall et al [30], 2008 McCann et al [31], 2009; Kearney et al [32], 2009 Passardi et al [36], 2022 Weaver et al [33], 2014 Xu et al [63], 2019	
**Supportive features**					
	Automated self-care advice	9 (22)		Chumbler et al [23], 2007 Coolbrandt et al [40], 2022 Kearney et al [28], 2006 Maguire et al [29], 2005 Maguire et al [71], 2015 McCall et al [30], 2008 McCann et al [31], 2009; Kearney et al [32], 2009 Weaver et al [33], 2014 Yap et al [37], 2013	
	Clinician alerts	9 (22)		Besse et al [34], 2016 Coolbrandt et al [40], 2022 Kearney et al [28], 2006 Maguire et al [29], 2005 Maguire et al [71], 2015 McCann et al [31], 2009; Kearney et al [32], 2009 Mooney et al [26], 2014 Weaver et al [33], 2014 Yap et al [37], 2013	
	Clinician could view summary of responses	5 (12)		Coolbrandt et al [40], 2022 Harper et al [24], 2012 Kearney et al [28], 2006 Min et al [61], 2014 Nordhausen et al [42], 2022	
	Informational modules	2 (5)		Çınar et al [21], 2021 Passardi et al [36], 2022	
	Module allowing communication with clinicians	1 (2)		Çınar et al [21], 2021	
	Patients received response summaries	2 (5)		McCall et al [30], 2008 Xu et al [63], 2019	
	Relaxation reminders	1 (2)		Çınar et al [21], 2021	
	None mentioned	23 (56)		Aigner et al [22], 2016 Badr et al [45], 2010; Badr et al [46], 2013; Stephenson et al [47], 2018; Belcher et al [48], 2011; Pasipanodya et al [49], 2012 Otto et al [50], 2015 Carson et al [52], 2021 Dasch et al [53], 2010 Dunsmore et al [72], 2023 Hachizuka et al [35], 2010 Kim et al [60], 2016 Langer et al [25], 2018 LeBaron et al [38], 2022; LeBaron et al [39], 2023 Lee et al [41], 2023 Lim et al [64], 2022 Pinto et al [54], 2021 Ratcliff et al [55], 2014 Schuler et al [43], 2023 Shiyko et al [68], 2014; Shiyko et al [67], 2019 Solk et al [56], 2019; Phillips et al [57], 2020; Auster-Gussman et al [58], 2022; Welch et al [65], 2023; Whitaker et al [66], (2023) Steffen et al [69], 2018; Steffen et al [70], 2020 Stone et al [59], 2016 Sztachańska et al [62], 2019 van den Berg et al [27], 2022 van Roozendaal et al [44], 2023	

^a^Multiple options possible per study.

Out of the 41 studies, 22 (54%) studies [25,34-40,43-51,54-58,61,63,65-70,72] applied signal-contingent sampling (ie, prompting respondents to complete the questionnaire) and 6 (15%) studies [21,30,33,53,59,62] applied interval-contingent sampling (ie, instructing respondents to complete the questionnaire at certain intervals), while 15 (37%) studies [22-24,26-29,31,32,36,41,52,60,64,71] did not specify the sampling method. Furthermore, 6 (15%) studies used event-contingent sampling on top of the other sampling methods; of these, 4 (67%) studies [29-32,38,39] instructed patients to complete the assessment when experiencing adverse events, 1 (17%) study [35] required the patients to assess when rescue medication was taken, and 1 (17%) study [43] prompted patients when a physiologically measured stress threshold was reached. Out of 22 signal-contingent sampling studies, 13 (59%) [25,34-37,43,48-51,61,63,69,70,72] prompted patients at fixed times, with times between prompts ranging from 3 to 24 hours. Moreover, 36% (8/22) of the studies [35,42,44-47,54-58,65-68] prompted patients at random times, of which 5 (62%) [35,44-47,55,67,68] randomly prompted within a fixed time block (eg, between 9 AM and midnight). Minimum time intervals between randomly timed prompts ranged from 30 minutes to 3 hours [45-47,54-58].

Of the 41 studies, 7 (17%) [31-33,52,54-58,65,66,71] had multiple data collection periods for each patient. While the most common data collection period lengths were 7 days [28,35,48-54,71,72] and 14 days [22,25,29,31,32,45-47,62,67,68], ranging from 1 to 336 days [42,77], 10 (24%) studies [23,24,26,36,38-40,42,44,55,60] mentioned differing study lengths for each patient (eg, based on patients’ next chemotherapy visit) [55].

#### System Characteristics

Data collection devices and software varied substantively in the included studies (n=41), with 11 (27%) studies [21,25,27,40,43,44,51,54,56-58,61,63,65,66] using smartphones, 1 (2%) study using smartwatches [38,39], and 8 (20%) studies [22,24,28,30,35,45-47,55,67,68] using handheld computers for self-report assessments. Other studies used basic telephone and SMS text messaging functionality [26,34,37,41,51,52], internet functionality [48-50,53,56-59,65,66,69,70,72], and used a specifically developed device [23,38,39]. A total of 19 (46%) studies [21,26,27,34,37,40,41,43,44,48-54,56-59,61,63] used patients’ devices, whereas 12 (29%) studies [22-24,28,30,33,35,38,39,42,45-47,55,67,68] provided devices to patients.

Different types of software were used, including smartphone apps [21,25,27,36,40,43,44,54,60,61,63,64], browser-based surveys [48-50,53,56-59,63,65,66,69,70,72], SMS text messaging [34,37,51], interactive voice responding systems [26,34,41,52], and other specifically developed software applications [22,23,28,29,31-33,38,39,42,55,71].

#### Questionnaire Length

Some studies (7/41, 17%) [25,26,38-40,43,45-50,65-68] used conditional items that were presented when a certain response was given to previous items and different questionnaires depending on the timing of the prompt (eg, the use of morning prompts to assess sleep quality [55,56]). Most studies (20/41, 49%) [21-25,27-29,31-35,37-40,42-47,51,52,55-61,63,65-68] had questionnaire lengths ranging between 1 and 20 items, with the longest being 84 items (including conditional items) [48-50]. Several studies (13/41, 32%) [21,23,28-33,36,40,51,63,64,71] did not provide complete information on the number of items.

#### Supportive Features

Of 41 studies, 17 (41%) [21,23,24,26,28-34,36,37,40,42,61,63,71] provided supportive features; 9 (22%) studies [23,28-33,37,40,71] offered automated self-care advice to patients based on their responses directly after response submission, for instance, offering advice for managing reported symptoms, with severe symptoms triggering advice to contact a health care professional [73]. Also, 9 (22%) studies [26,28,29,31-34,37,40,71] automatically contacted health care professionals based on symptom severity (ie, clinician alerts). Some studies (2/41, 5%) [31,32,71] differentiated between different severities to indicate varying levels of need for immediate intervention (eg, amber and red alerts). A total of 6 (15%) studies [28,29,31-33,37,71] combined automated self-care advice and clinician alerts. One study [26] alerted clinicians based on responses given on domains other than physical symptoms, namely psychological variables (ie, depressive mood and anxiety) and distress caused by symptoms. Other supportive features included providing the opportunity to clinicians to view a summary or visualization of responses given by the patient [24,28,40,42,61] and providing patients with informational modules [21,36], modules allowing communication with clinicians [21,36], response summaries [30,63], and relaxation reminders [21].

### Study-Reported Findings

#### Findings Concerning Methodological Evaluations

Intensive longitudinal methods that sampled once daily [23,26-28,30,37,40,42] or multiple times per day [33-35,38,43,56] were deemed feasible and acceptable for patients. These findings applied to various system characteristics, such as interactive voice response and SMS text messaging systems [26,34,37] and smartphone apps [27,40,43]. Compliance decreased over time in a 90-day study [61], with higher compliance among unemployed women. Patients believed in the method’s ability to improve symptoms [29], symptom management [28,71], and communication with clinicians [71]. Moreover, patients had positive views on the usability of the methods [26,30,34,35,56,71] and felt reassured by using them [29,33].

Health care professionals had a positive view of the methods [71] and found them reassuring for patients, especially during out of hours [33], and clinically useful [26,30,37]. In addition, health care professionals thought that the methods could be helpful aids in timely interventions [29] and for assessing [28] and managing symptoms [28,29]. However, one study [24] reported that quality of life data was not used for making treatment decisions, and other studies [26,42,64] reported that clinicians rarely contacted the patients after receiving clinical alerts or monitored their responses. In one study [71], health care professionals mentioned that reduced complexity of the system was needed to promote its utility.

Some studies (5/41, 12%) [34,36,41,51,59] compared intensive longitudinal methods with other scales and found agreement between the methods, such as depression ratings and Patient Health Questionnaire-9 [60,78]. One study [27] found a lack of agreement between the intensive methods and the Short Form Health Survey [79], but this concordance improved with higher compliance rates.

#### Findings Concerning Prevalence and Covariability of Constructs

Several studies (16/41, 39%) examined the prevalence and covariability of constructs ranging across multiple topics. For instance, 7 studies [25,45,46,48-50,63] reported findings related to the social dynamics between patients and their partners. One study [45] found greater reports of relationship interference when patients experienced more pain and lower arousal mood. Moreover, partners were more likely to provide support when patients experienced more tiredness and less active mood resulting from pain [45]. Another study on this topic [48] found that partners’ reports of support provision were positively associated with feelings of relationship intimacy reported by patients.

Overall, studies investigated various topics such as physical activity, affect, and physical symptoms. For instance, studies [54,65] showed associations between sedentary behavior, affective valence, and fatigue at different time points, analog to other studies [57,66] that found within-person associations between physical activity and same-day affect, fatigue, pain, and others.

#### Findings Concerning the Intensive Methods as an Intervention

Of the 41 studies, 7 (17%) [21,23,26,31,32,34,71] investigated the impact of intensive longitudinal methods as an intervention tool to improve symptoms, for instance, by providing automated self-care advice to patients or alerting clinicians when a certain symptom threshold was reached [71]. Patients in the intervention groups reported lower distress [21], lower fatigue, and higher levels of hand-foot syndrome [32] than those in the control groups. Patient-reported benefits included improved communication with health care professionals and symptom management and reassurance that symptoms were being monitored at home [31]. After the intervention, patients reported increased quality of life [21,23], lower anxiety and drowsiness, lower pain [34], and higher self-care efficacy [71] than at the baseline. One study using clinician alerts [26] found no improvements in symptom severity, explained by clinicians rarely contacting patients after alerts.

### Response-Related Results

Of the 41 studies, 21 (51%) [22-26,33,34,37,40,43-50,52-54,56-58,63,65-67,69-71] reported participation rates ranging from 23.6% to 90.3% (mean 52.9, SD 3.4; Table 3; Multimedia Appendix 4) [21-72]. Overall, 17 (41%) studies [23,25,26,30,34,35,37-40,43,44,51,55,61,63,64,71] reported attrition rates, ranging from 0% to 56.9% (mean 19.7%, SD 17.7%). Furthermore, 19 (46%) studies [22,27,28,31-33,36,45-50,52-54,56-60,62,65-70,76] provided other attrition indicators, while 29 (71%) studies [22-27,33-35,40,42,43,45-47,50-67,69,70,72] reported compliance rates ranging from 44.2% to 98% (mean 74.9%, SD 16.4%).

**Table 3:** Response-related results of the included studies (n=41).

Results and characteristics		Frequency of studies, n (%)	Reference, year
**Participation rate**			
	23%-25%	3 (7)	Coolbrandt et al [73], 2021 Solk et al [56], 2019; Phillips et al [57], 2020; Auster-Gussman et al [58], 2022; Welch et al [65], 2023; Whitaker et al [66], 2023 van Roozendaal et al [44], 2023
	26%-50%	8 (20)	Aigner et al [22], 2016 Belcher et al [48], 2011; Pasipanodya et al [49], 2012 Otto et al [50], 2015 Carson et al [52], 2021 Dasch et al [53], 2010 Langer et al [25], 2018 Maguire et al [71], 2015 Xu et al [63], 2019 Yap et al [37], 2013
	51%-75%	4 (10)	Badr et al [45], 2010; Badr et al [46], 2013; Stephenson et al [47], 2018; Pinto et al [54], 2021 Schuler et al [43], 2023 Weaver et al [33], 2014
	76%-90%	6 (15)	Besse et al [34], 2016 Chumbler et al [23], 2007 Harper et al [24], 2012 Mooney et al [26], 2014 Shiyko et al [68], 2014; Shiyko et al [67], 2019 Steffen et al [69], 2018; Steffen et al [70], 2020
	Not mentioned	19 (46)	Otto et al [50], 2015 Cai et al [51], 2020 Çınar et al [21], 2021 Dunsmore et al [72], 2023 Hachizuka et al [35], 2010 Kearney et al [28], 2006 Kim et al [60], 2016 LeBaron et al [38], 2022; LeBaron et al [39], 2023 Lee et al [41], 2023 Lim et al [64], 2022 Maguire et al [29], 2005 McCall et al [30], 2008 McCann et al [31], 2009; Kearney et al [32], 2009 Min et al [61], 2014 Nordhausen et al [42], 2022 Passardi et al [36], 2022 Ratcliff et al [55], 2014 Stone et al [59], 2016 Sztachańska et al [62], 2019 van den Berg et al [27], 2022
**Attrition rate**			
	0%-25%	12 (29)	Cai et al [51], 2020 Coolbrandt et al [40], 2022 Hachizuka et al [35], 2010 Harper et al [24], 2012 Langer et al [25], 2018 Min et al [61], 2014 Mooney et al [26], 2014 Ratcliff et al [55], 2014 Schuler et al [43], 2023 van Roozendaal et al [44], 2023 Xu et al [63], 2019 Yap et al [37], 2013
	26%-57%	6 (15)	Besse et al [34], 2016 Chumbler et al [23], 2007 LeBaron et al [38], 2022; LeBaron et al [39], 2023 Lim et al [64], 2022 Maguire et al [71], 2015 McCall et al [30], 2008
	Other indicators mentioned	18 (44)	Aigner et al [22], 2016 Badr et al [45], 2010; Badr et al [46], 2013; Stephenson et al [47], 2018; Belcher et al [48], 2011; Pasipanodya et al [49], 2012 Otto et al [50], 2015 Carson et al [52], 2021 Dasch et al [53], 2010 Kearney et al [28], 2006 Kim et al [60], 2016 Lee et al [41], 2023 McCann et al [31], 2009; Kearney et al [32], 2009 Passardi et al [36], 2022 Pinto et al [54], 2021 Shiyko et al [68], 2014; Shiyko et al [67], 2019 Solk et al [56], 2019; Phillips et al [57], 2020; Auster-Gussman et al [58], 2022; Welch et al [65], 2023; Whitaker et al [66], 2023 Steffen et al [69], 2018; Steffen et al [70], 2020 Stone et al [59], 2016 Sztachańska et al [62], 2019 van den Berg et al [27], 2022 Weaver et al [33], 2014
	None mentioned	5 (12)	Otto et al [50], 2015 Çınar et al [21], 2021 Dunsmore et al [72], 2023 Maguire et al [29], 2005 Nordhausen et al [42], 2022
**Compliance rate**			
	44%-60%	6 (15)	Otto et al [50], 2015 Kim et al [60], 2016 Min et al [61], 2014 Ratcliff et al [55], 2014 Schuler et al [43], 2023 van den Berg et al [27], 2022
	61%-80%	10 (24)	Aigner et al [22], 2016 Badr et al [45], 2010; Badr et al [46], 2013; Stephenson et al [47], 2018 Besse et al [34], 2016 Carson et al [52], 2021 Coolbrandt et al [40], 2022 Dunsmore et al [72], 2023 Mooney et al [26], 2014 Pinto et al [54], 2021 Shiyko et al [68], 2014; Shiyko et al [67], 2019 Xu et al [63], 2019
	81%-100%	13 (32)	Cai et al [51], 2020 Chumbler et al [23], 2007 Dasch et al [53], 2010 Hachizuka et al [35], 2010 Harper et al [24], 2012 Langer et al [25], 2018 Lim et al [64], 2022 Nordhausen et al [42], 2022 Solk et al [56], 2019; Phillips et al [57], 2020; Auster-Gussman et al [58], 2022; Welch et al [65], 2023; Whitaker et al [66], 2023 Steffen et al [69], 2018; Steffen et al [70], 2020 Stone et al [59], 2016 Sztachańska et al [62], 2019 Weaver et al [33], 2014
	Other indicators mentioned	6 (15)	Belcher et al [48], 2011; Pasipanodya et al [49], 2012 LeBaron et al [38], 2022; LeBaron et al [39], 2023 Lee et al [41], 2023 Passardi et al [36], 2022 van Roozendaal et al [44], 2023 Yap et al [37], 2013
	Not mentioned	6 (15)	Çınar et al [21], 2021 Kearney et al [28], 2006 Maguire et al [29], 2005 Maguire et al [71], 2015 McCall et al [30], 2008 McCann et al [31], 2009; Kearney et al [32], 2009
**Monetary incentives**			
	Amount based on the number of completed assessments	6 (15)	Badr et al [45], 2010; Badr et al [46], 2013; Stephenson et al [47], 2018 Belcher et al [48], 2011; Pasipanodya et al [49], 2012 Otto et al [50], 2015 Langer et al [25], 2018 Pinto et al [54], 2021 Ratcliff et al [55], 2014 Steffen et al [69], 2018; Steffen et al [70], 2020
	Fixed amount	5 (12)	Cai et al [51], 2020 Carson et al [52], 2021 Stone et al [59], 2016 LeBaron et al [38], 2022; LeBaron et al [39], 2023 Solk et al [56], 2019; Phillips et al [57], 2020; Auster-Gussman et al [58], 2022; Welch et al [65], 2023; Whitaker et al [66], 2023
	None provided	2 (5)	Min et al [61], 2014 van den Berg et al [27], 2022
	Not specified	28 (68)	Aigner et al [22], 2016 Otto et al [50], 2015 Besse et al [34], 2016 Chumbler et al [23], 2007 Çınar et al [21], 2021 Coolbrandt et al [40], 2022 Dasch et al [53], 2010 Dunsmore et al [72], 2023 Hachizuka et al [35], 2010 Harper et al [24], 2012 Kearney et al [28], 2006 Kim et al [60], 2016 Lee et al [41], 2023 Lim et al [64], 2022 Maguire et al [29], 2005 Maguire et al [71], 2015 McCall et al [30], 2008 McCann et al [31], 2009; Kearney et al [32], 2009 Mooney et al [26], 2014 Nordhausen et al [42], 2022 Passardi et al [36], 2022 Schuler et al [43], 2023 Shiyko et al [68], 2014; Shiyko et al [67], 2019 Sztachańska et al [62], 2019 van Roozendaal et al [44], 2023 Weaver et al [33], 2014 Xu et al [63], 2019 Yap et al [37], 2013

Overall, 32% (13/41) of the studies provided monetary incentives, of which 8 (62%) studies [25,45-50,54,55,59,69,70] based attainable monetary amounts on the number of completed assessments, while 5 (38%) [38,39,51,52,56-58,65,66,72] provided patients with fixed amounts. Attainable monetary amounts ranged from US $40 to $200.

### Barriers and Facilitators

Most studies reported the barriers and facilitators regarding the implementation of their methods in research or clinical practice (Table 4), either related to the person with cancer or the methods themselves. Some facilitating person-related factors included having confidence in using technology systems [31,56] and recognizing its clinical benefits [28,30,60]. Some person-related barriers were lack of smartphone ownership [40,61] and discomfort with technology [30,45-47,71]. However, inexperience with technology generally did not impact success with the study technologies [25,28,31,35]. However, smartphone users had higher compliance during an SMS protocol than basic phone users [37].

**Table 4:** Barriers and facilitators for the implementation of the method in practice and for research purposes, as stated by the papers’ authors or extracted from the reported results.

Themes	Facilitators	Barriers
Factors related to the person with breast or lung cancer	*Confidence* in their abilities to use technology systems [31,56] Overall *preference for online diary* compared with paper diary [62] Smartphone *users* had higher compliance than basic phone users [37] *Recognize the clinical benefits* of using technology systems to report symptoms [28-30,42,60] and weigh these benefits against assessment burden [43] *Willingness of patients* [30,42] Patient *perceptions on the relevance* of the study to their needs [29] *Sex, age, and diagnosis* did not impact compliance [42,43]; excluded participants appeared similar to the included participants [44] *(Belief that) data are used* by clinicians [30,42,73]	*Lack of interest or motivation* to participate can lead to small sample size [22] and lower compliance [42] *Time constraints* affect participation rate and compliance [22,42,45,46] *Symptoms and* side effects due to (advanced stage) illness and treatment may cause increased burden during study period, problems with pressing buttons, lower participation and compliance rates, and bias due to missing data [38,42,45-47,51,55,56,69] Men were more likely to not use monitoring than women [26] *Not owning a* smartphone prevents certain patients from using the monitoring system and thus participating in the study [61,73] *Inexperience and discomfort about using the technology system* at start of the study period; particularly, *older adults* were less likely to participate [30,45-47,71] *Caregiver status not easily verifiable through electronic health record*, disrupting eligibility screening [38] Health care *professionals had doubts about the ability of patients* to complete electronic assessments [42] Some patients barely wearing or averse to wearing the study device [38,43] *Dyad studies require informed consent from patient and caregiver*, leading to logistical challenges [38] Difficulties *remembering experiences with using the system* after the study period [31]
Factors related to the method	*Use of single items for constructs* to shorten questionnaire [39,48,58,69] reduces burden, improves adherence [39], and gives room for measurement of multiple constructs, possibly reducing reactivity to a single construct [69] *Tailoring of sampling schedule to population of interest*, for example, limiting the frequency of assessments, to not overburden [67] or providing a broad enough window to respond in [53], possibly prompting the participant a second time if unanswered [55] *Reminders or prompts*, including the option to tailor reminder schedules and contact by the researcher, might improve adherence [21,31,36,54,57,58,61] Ability to use patients’ *personal smartphones* [34,57], making the need for study visits to receive a specialized electronic study device obsolete [27,34,56] and providing a nonburdensome means to study individuals in their natural environment [27,34] *Possibility to combine EMA^a^ prompting with passive monitoring* through high-grade commercially available devices [43,57] *Using electronic devices over paper-and-pencil* alternatives does not impact attrition [32] *Portability* of mobile phones enables daily assessments [60], while smartwatches can enhance acceptability [38] *Facial emotions scale* demands less cognitive effort, is less of a burden, and makes responding more enjoyable [60] *“Unsure” response option* can improve data quality when patients are confused with a question [38] *Simple questionnaire and system design* for an easier patient experience [29,31,42] Option to report *additional information* after structured questionnaire for a better patient experience (eg, additional symptoms and having preexisting conditions) [31] *More time explaining how to respond* correctly to SMS response system can improve the quality of responding when the response format is expected to be difficult [37] *Standardized protocol checklist* for researchers to streamline deployment installation [38] *Providing participants with handouts* before the study period, including frequently asked questions and contact information in case of difficulties in using system [25] *Easy and fast access to PROMs^b^ and gathered data*, for example, by the integration of monitoring system into the electronic patient, likely leads more uptake in clinical settings [30,42,73] and makes IT support crucial [42] *Cloud services system* improves the ability to securely off-load and store data in real time [38] *Reducing time delays between consent and deployment* can mitigate attrition and accommodate the dynamic clinical status of patients [38] *Iterative deployments* can improve setting up and removing the system [38] *Personal support by research assistant* is appreciated by patients [42] and might improve adherence [44]	*Single item constructs bring psychometric limitations* [39] *Empty battery or low battery life*, possibly leading to device memory loss and missing data [38,45,46,51] *Turned off phones or patients not wearing smartwatches* leading to missing data [38,51] *Transmission or pairing errors* [33,38,42,51] can lead to frustrations [38] Bugs in code *to monitor smartwatches* [38] *Incompatibility issues* possible between smartphones’ display specifications and the used app [61] *Synchronization problems* related to automatic Android updates leading to inconsistent timing of EMA prompts [38] *Poor reception* at home, for example, in rural areas [31,33], could cause necessity to switch SIM providers [33] *Monitoring requires time and manpower* in a context with high clinician time constraints [37,42,71], possibly leading to fewer calls after clinician alerts [26], or lack of using monitoring results by clinical staff and trial investigators [42,64] *Dependency of the implementation on* health care *professionals*, who are difficult to motivate to break the status quo [42] *Vast amount of data* can be burdensome to clinicians [60] *False-positive clinician alerts* due to errors in responding and transmission problems [33,37] *Self-care information* not always read by patients [71] Compliance to *time-blocked random signals* may be affected by participants waking up late or going to bed early [46] *Developing EMA schemes can be challenging* when taking participant burden into account [39] *Content irrelevant to patient* could cause dissatisfaction [37]; clinical monitoring measures should be tailored to their needs [42] *24-hour recall may not be appropriate* to measure all symptoms [41] *Unclear instructions* on when to complete event-contingent assessment can cause confusion among participants [38] *Technical changes are complex and require time* to test and implement, but are often underestimated by clinical team [38] In comparative trials, *electronic diary might bias patients* toward better self-management due to increased awareness and daily requirement to enter data [64] *Interruption of monitoring assessment* (eg, due to diagnostics or therapy) [42] *Rapid clinical staff turnover* [42]
Other factors	COVID-19 *pandemic* [38,42]	—^c^

^a^EMA: ecological momentary assessment.

^b^PROM: patient-reported outcome measure.

^c^Not applicable.

Some facilitating method-related factors included the ability to tailor sampling schedules to the population of interest [53,55,67] and the option to use reminders [21,31,36,54,57,58,61]. Some barriers included technical issues such as empty batteries leading to memory loss and missing data [38,45,46,51] and false-positive clinician alerts due to faulty responding and transmission problems [33,37]. All these factors were associated with improvements in participation and compliance rates, user-experience, patient burden, quality of responses, time requirements for researchers, and adoption in clinical settings [21,22,25-34,36-38,40,42-48,51,55-58,60,61,67,69,71].

## Discussion

### Principal Findings

Intensive longitudinal methods with daily electronic assessments have been used among people with breast or lung cancer at different disease stages. The methods involved 1-6 assessments per day to study a wide range of experiences in daily life, primarily physical and psychological symptoms. Some studies integrated supportive features within the longitudinal assessments. For most studies, compliance and attrition rates were acceptable, although many studies lacked complete methodological reporting. Few studies focused on patients in the advanced stage of disease. We identified the barriers and facilitators for using these methods, related to both the person with cancer and the method itself.

Our review highlights the promise of intensive longitudinal methods to provide unique insights into the daily lives of people living with cancer. Importantly, these methods generally seem feasible and acceptable among patients with breast or lung cancer, supported by positive patient and health care professional experiences, along with compliance and attrition rates indicating acceptable amounts of missing data. These findings were true for different methodological approaches, such as studies that assessed patients once or multiple times daily. Moreover, these methods demonstrate flexibility as they were used to address an array of objectives, such as exploring within-person symptom associations [55] or communication patterns in dyads [63].

Before widespread implementation of these intensive methods in oncology research and practice, several of our findings encourage further investigation into its feasibility and optimal study conditions. First of all, it is striking that response- and methodology-related reporting was often incomplete or reported in different ways (eg, compliance rates and amount of questionnaire items). Standardized reports of this information are critical to inform optimal methodological choices in future studies or clinical procedures, as poor choices can lead to additional patient burden and missing data. Due to the unstandardized reporting by many included studies, comparisons in response-related results between studies with different methodological features were not possible in this review. Yet, such comparisons are particularly important when using intensive sampling methods in populations who are already susceptible to increased disease-related burden. In addition, several identified factors need further exploration to enhance the implementation of intensive longitudinal methods with daily electronic assessments in research and practice, for example, participants’ feelings of inexperience and discomfort with technology leading to a lower likelihood to participate in the study [30,45-47,71]. Finally, low participation rates of the included studies indicate participant recruitment to be difficult, and sample sizes were often small. This is a major barrier for research, as it could lead to sampling bias, for instance, through self-selected sampling of people more confident or experienced in using electronic systems. Subsequently, this could limit the validity of study findings.

Our review identified understudied areas that prevent gaining a complete understanding of people with breast or lung cancer and their daily experiences. First, several populations of people with breast or lung cancer are currently underrepresented in intensive longitudinal method studies, which significantly limits the generalizability of findings for these populations, including findings on the feasibility of these methods. For instance, of the 41 studies, only 4 (10%) were conducted in people with lung cancer specifically, 6 (15%) studies were conducted in people with stage IV cancer specifically, 1 (2%) study was conducted in a low-income country, and only 1 (2%) study included 1 male participant with breast cancer. Second, although the study objectives varied widely, studies predominantly focused on the aspects of physical health, such as pain, or had rather clinical views on psychological constructs by focusing on depression and anxiety. Only one included study [62] covered experiences from spiritual or existential quality of life domains, which is remarkable because these experiences generally have increasing value at the end of life [5,6]. Furthermore, although ESMs offer the potential for linking patient experiences with concurrent contexts (eg, where the patient is and what they are doing) [12], these contextual aspects remain understudied among people with breast or lung cancer. A broader focus encompassing different domains and contexts is needed to gain a more comprehensive understanding of patients’ quality of life and well-being, ultimately enabling the improvement of patient-centered care.

### Implications for Practice and Research

On the basis of our findings, we provide several recommendations for practice and research. First, applying existing reporting guidelines for EMAs, such as those synthesized by Liao et al [80], can improve transparency and consistency in reporting for intensive longitudinal studies in oncology. Their checklist serves as a starting point to fulfill recommended reporting criteria, such as reporting the use of prompts and complete questionnaire information [81]. This will allow future researchers to accurately explore the effects of study features on response-related results.

Second, addressing implementation factors highlighted in this review can be achieved through simple solutions, such as providing clear instructions, training on the use of the methods, and emphasizing the importance of the study to increase patient motivation and confidence [25,28-31,37,56,60]. Moreover, extensive pretesting such as conducting a pilot study is essential to uncover any technical issues that may arise.

Third, it is essential to determine optimal conditions for using intensive longitudinal methods with daily electronic assessments in people with cancer, such as ideal sampling schemes for the feasible measurement of specific constructs [82,83]. Studies should focus on populations at an increased risk for symptom burden, such as those with advanced stage cancer [84,85]. Furthermore, the use of supportive features such as automated feedback and clinician alerts needs more investigation to explore how it is optimally implemented in routine clinical practice for the best possible outcomes. Moreover, it is recommended to develop measures to examine the quality of responses provided by patients [86], as these could be influenced by cancer and its treatment (eg, through cognitive impairment).

Fourth, future studies among patients with breast and lung cancers could broaden their focus to encompass more nonclinical psychological or spiritual-existential topics and contextual factors. This approach could yield novel insights into the interplay between physical functioning and other aspects of well-being and how they vary in different contexts [8]. Researchers could look to other populations of people living with or beyond cancer to further inform on the possibilities of these methods. For example, studies involving survivors of cancer could have a less clinical focus due to living past the treatment stage. Future literature reviews of the use of daily methods among such populations would be greatly beneficial.

Finally, studies should further explore how multiple daily measurements compare with the same constructs as measured by the more commonly used patient-reported outcome measures in oncology, in which patients are expected to aggregate experiences over ≥1 weeks [87,88]. Such research could examine the ecological validity of these commonly used patient-reported outcome measures [59] and provide valuable insights for oncology research and practice regarding which experiences are more accurately measured on a more frequent basis.

### Strengths and Limitations

This scoping review followed a broad systematic search strategy in multiple databases, incorporating studies that used self-report methods to assess patients daily or multiple times a day. Consequently, it offers a comprehensive overview of the methods used to gain insight into the daily experiences of people with breast and lung cancers at various stages across different countries.

Nevertheless, this review has limitations. First, it is plausible that we missed studies that used different terms for their daily electronic self-report questionnaire than those used in our search string. However, the broadness of our search string minimized this risk, and we detected articles that reported on methods that could be classified as ESMs but were not identified by the previous review in 2019 [12]. Second, only 10% of data extraction was checked by a second reviewer, and none were compared during the updated search, introducing a slight possibility of inaccuracies. We consider this a minor risk, as we found no disagreements in the 10% data that we had checked.

### Conclusions

Intensive longitudinal methods using daily electronic assessments hold promise and can be feasible to provide unique insights into the daily lives of patients with breast or lung cancer. However, our findings encourage further research on the feasibility of determining optimal conditions for intensive monitoring, specifically in more advanced disease stages, and better adherence to standardized reporting guidelines. Moreover, considering a more multidimensional approach to the topics studied, especially beyond physical and psychopathological symptoms, will enhance the value of these methods, ultimately aiding in the improvement of patient-centered care in oncology.

## Appendix

Multimedia Appendix 1 Search terms.

Multimedia Appendix 2 Study and sample characteristics.

Multimedia Appendix 3 Content and design characteristics.

Multimedia Appendix 4 Response-related characteristics.

Multimedia Appendix 5 PRISMA Scoping Review Checklist.

## Abbreviations

EMA: ecological momentary assessment
EORTC-QLQ-C30: EORTC Core Quality of Life Questionnaire
ESM: experience sampling methods
PRISMA-ScR: Preferred Reporting Items for Systematic Reviews and Meta-Analyses extension for Scoping Reviews
