# Supplementary material for: Intensive Longitudinal Methods Among Adults With Breast or Lung Cancer: Scoping Review
Source: J Med Internet Res. 2024 Jun 12;26:e50224. doi: 10.2196/50224 (PMC11208836; doi:10.2196/50224)
Supplement: Multimedia Appendix 1 [file jmir_v26i1e50224_app1.doc]

**Appendix 1.** Search terms.

| 1.PubMed  (“neoplasms”[mesh] OR neoplasm*[tiab] OR cancer[tiab] OR oncolog*[tiab] OR cancer*[tiab] OR neoplas*[tiab] OR tumour*[tiab] OR tumor*[tiab] OR carcinom*[tiab] OR melanom*[tiab] OR lymphom*[tiab] OR leukemi*[tiab] OR malignan*[tiab] OR metasta*[tiab] OR carcinogen*[tiab] OR oncogen*[tiab] OR anticarcinogen*[tiab] OR sarcoma*[tiab] OR precancerous[tiab] OR paraneoplastic[tiab] OR neuroma*[tiab] OR blastoma*[tiab] OR meningioma*[tiab] OR lymphangioma*[tiab] OR lymphangiomyoma*[tiab] OR lymphangiosarcoma*[tiab] OR "hodgkin disease"[tiab] OR plasmacytoma*[tiab] OR carcinosarcoma*[tiab] OR hepatoblastoma*[tiab] OR mesenchymoma*[tiab] OR chordoma*[tiab] OR germinoma*[tiab] OR gonadoblastoma*[tiab] OR mesonephroma*[tiab] OR teratoma*[tiab] OR teratocarcinoma*[tiab] OR nsclc[tiab])  AND (“ecological momentary assessment”[mesh] OR “momentary assess*”[tiab] OR “experience sampling*”[tiab] OR “intensive longitudinal”[tiab] OR “daily diar*”[tiab] OR “symptom diar*”[tiab] OR “interaction record*”[tiab] OR “ambulatory assess*”[tiab] OR “mobile assess*”[tiab] OR “mobile interven*”[tiab] OR “event sampling”[tiab] OR “structured diary method*”[tiab] OR “real-time data capture”[tiab] OR “daily symptom monitoring”[tiab] OR “electronic diar*”[tiab] OR “structured diar*”[tiab] OR “computer diar*”[tiab]) |
| --- |
| 2.Embase  ('neoplasm'/exp OR neoplasm*:ab,kw,ti OR cancer:ab,kw,ti OR oncolog*:ab,kw,ti OR cancer*:ab,kw,ti OR neoplas*:ab,kw,ti OR tumour*:ab,kw,ti OR tumor*:ab,kw,ti OR carcinom*:ab,kw,ti OR melanom*:ab,kw,ti OR lymphom*:ab,kw,ti OR leukemi*:ab,kw,ti OR malignan*:ab,kw,ti OR metasta*:ab,kw,ti OR carcinogen*:ab,kw,ti OR oncogen*:ab,kw,ti OR anticarcinogen*:ab,kw,ti OR sarcoma*:ab,kw,ti OR precancerous:ab,kw,ti OR paraneoplastic:ab,kw,ti OR neuroma*:ab,kw,ti OR blastoma*:ab,kw,ti OR meningioma*:ab,kw,ti OR lymphangioma*:ab,kw,ti OR lymphangiomyoma*:ab,kw,ti OR lymphangiosarcoma*:ab,kw,ti OR ‘hodgkin disease’:ab,kw,ti OR plasmacytoma*:ab,kw,ti OR carcinosarcoma*:ab,kw,ti OR hepatoblastoma*:ab,kw,ti OR mesenchymoma*:ab,kw,ti OR chordoma*:ab,kw,ti OR germinoma*:ab,kw,ti OR gonadoblastoma*:ab,kw,ti OR mesonephroma*:ab,kw,ti OR teratoma*:ab,kw,ti OR teratocarcinoma*:ab,kw,ti OR nsclc:ab,kw,ti )  AND (‘ecological momentary assessment’/exp OR ‘momentary assess*’:ab,kw,ti OR ‘experience sampling*’:ab,kw,ti OR ‘intensive longitudinal’:ab,kw,ti OR ‘daily diar*’:ab,kw,ti OR ‘symptom diar*’:ab,kw,ti OR ‘interaction record*’:ab,kw,ti OR ‘ambulatory assess*’:ab,kw,ti OR ‘mobile assess*’:ab,kw,ti OR ‘mobile interven*’:ab,kw,ti OR ‘event sampling’:ab,kw,ti OR ‘structured diary method*’:ab,kw,ti OR ‘real-time data capture’:ab,kw,ti OR ‘daily symptom monitoring’:ab,kw,ti OR ‘electronic diar*’:ab,kw,ti OR ‘structured diar*’:ab,kw,ti OR ‘computer diar*’:ab,kw,ti) |
| 3.ProQuest: PsycINFO  [STRICT](TI,AB,SU(neoplasm*) OR TI,AB,SU(cancer) OR TI,AB,SU(oncolog*) OR TI,AB,SU(cancer*) OR TI,AB,SU(neoplas*) OR TI,AB,SU(tumour*) OR TI,AB,SU(tumor*) OR TI,AB,SU(carcinom*) OR TI,AB,SU(melanom*) OR TI,AB,SU(lymphom*) OR TI,AB,SU(leukemi*) OR TI,AB,SU(malignan*) OR TI,AB,SU(metasta*) OR TI,AB,SU(carcinogen*) OR TI,AB,SU(oncogen*) OR TI,AB,SU(anticarcinogen*) OR TI,AB,SU(sarcoma*) OR TI,AB,SU(precancerous) OR TI,AB,SU(paraneoplastic) OR TI,AB,SU(neuroma*) OR TI,AB,SU(blastoma*) OR TI,AB,SU(meningioma*) OR TI,AB,SU(lymphangioma*) OR TI,AB,SU(lymphangiomyoma*) OR TI,AB,SU(lymphangiosarcoma*) OR TI,AB,SU("hodgkin disease") OR TI,AB,SU(plasmacytoma*) OR TI,AB,SU(carcinosarcoma*) OR TI,AB,SU(hepatoblastoma*) OR TI,AB,SU(mesenchymoma*) OR TI,AB,SU(chordoma*) OR TI,AB,SU(germinoma*) OR TI,AB,SU(gonadoblastoma*) OR TI,AB,SU(mesonephroma*) OR TI,AB,SU(teratoma*) OR TI,AB,SU(teratocarcinoma*) OR TI,AB,SU(nsclc))  AND (TI,AB,SU(“momentary assess*”) OR TI,AB,SU(“experience sampling*”) OR TI,AB,SU(“intensive longitudinal”) OR TI,AB,SU(“daily diar*”) OR TI,AB,SU(“symptom diar*”) OR TI,AB,SU(“interaction record*”) OR TI,AB,SU(“ambulatory assess*”) OR TI,AB,SU(“mobile assess*”) OR TI,AB,SU(“mobile interven*”) OR TI,AB,SU(“event sampling”) OR TI,AB,SU(“structured diary method*”) OR TI,AB,SU(“real-time data capture”) OR TI,AB,SU(“daily symptom monitoring”) OR TI,AB,SU(“electronic diar*”) OR TI,AB,SU(“structured diar*”) OR TI,AB,SU(“computer diar*”)) |
