# Supplementary material for: Intensive Longitudinal Methods Among Adults With Breast or Lung Cancer: Scoping Review
Source: J Med Internet Res. 2024 Jun 12;26:e50224. doi: 10.2196/50224 (PMC11208836; doi:10.2196/50224)
Supplement: Multimedia Appendix 2 [file jmir_v26i1e50224_app2.docx]

**Multimedia Appendix 2.** Study and sample characteristics

| **First author, year, country** | **Study aims** | **Study design** | **Sample characteristics** | **Main findings** |
| --- | --- | --- | --- | --- |
| Aigner, 2016, USA | relation of pain to smoking and quit attempts in patients with pain during smoking cessation program | Observational: Electronic daily diary approach | • **Participants:** Mixed cancer populations (incl. breast and lung) • **Stage:** not mentioned • **Sample size:** N = 34 (Breast = 13, Lung = 10) • **Age:** M = 51.94 (SD = 10.30) • **Female:** 55.9% • **Comparison:** none | There was a positive within-person relation between pain and smoking. People with higher average pain were less likely to have days without smoking. |
| Badr, 2010; Badr, 2013; Stephenson, 2018 (USA) | • **Badr et al. (2010):**  relation between romantic relationships and pain • **Badr et al. (2013):**  trajectory of partner constraints, relation with patient affect and avoidance of cancer-related disclosure • **Stephenson et al. (2018):** associations between pain intensity and analgesic use | Observational: Electronic daily diary study | • **Participants:** Breast cancer, and their partners • **Stage:** IV • **Sample size:** N = 59 • **Age:** M = 49.38 (SD = 10.76) • **Female:** 100% • **Comparison:** none | **• Badr et al. (2010):** Greater relationship interference was reported when patients experienced more pain and less aroused mood. Tired mood during the day mediated the association between morning pain and evening ratings of received emotional and physical support. Partners were more likely to provide support when patients experienced more tired and less active mood resulting from pain. **• Badr et al. (2013):**  Partners' social constraints carried over from one day to the next, but patients' avoidance of discussing cancer-related concerns did not. When partners engaged in more social constraints one day, patients reported greater negative affect the following day. **• Stephenson et al. (2019):**  Between-person, pain was positively associated with prescription and ingestion of analgesics. Patients who typically experienced low pain tended to increase their medication use at times when their pain was higher than usual. |
| Belcher, 2011, USA | relation of patient support receipt and spouse support provision with daily intimacy, shortly after breast surgery | Observational: Electronic daily diary study | • **Participants:** Breast cancer, and their partners • **Stage:** 0-IIIa • **Sample size:** N = 54 • **Age:** M = 53 (SD = 9.7; couple) • **Female:** 100% • **Comparison:** none | Spouses' reports of support provision were positively associated with feelings of relationship intimacy by patients. The inter-partner effects of support receipt and support provision were also found to be beneficial for spouses' daily feelings of intimacy. |
| Pasipanodya, 2012, USA | influence of social constraints on daily event sharing, and individual and relationship well-being in couples, shortly after breast surgery | Observational: Electronic daily diary study | • **Participants:** Breast cancer, and their partners • **Stage:** 0-IIIa • **Sample size:** N = 45 • **Age:** M = 52.4 (SD = 9.9) • **Female:** 100% • **Comparison:** none | Patient-reported social constraints were negatively linked to patient sharing of both cancer-related and other important daily events. Higher patient and spouse perceptions of social constraints predicted both reduced daily individual and relationship well-being. |
| Otto, 2015, USA | relation between capitalization processes and daily intimacy and well-being, shortly after breast surgery | Observational: Electronic dialy diary methodology | • **Participants:** Breast cancer, and their partners • **Stage:** 0-IIIa • **Sample size:** N = 99 • **Age:** M = 52.27 (SD = 10.43) • **Female:** 100% • **Comparison:** none | Capitalization attempts were positively associated with feelings of intimacy. Perceived partner responsiveness to capitalization attempts was positively associated with the sharer’s feelings of intimacy and negatively with the sharer’s negative affect. When the partner was the sharer, perceived responsiveness of the patient was positively associated with positive affect. |
| Besse, 2016, the Netherlands | feasibility of using SMS and interactive voice response to improve pain management | Interventional: Feasibility of interactive voice response in combination with SMS alerts, with pre- and postmeasurements | • **Participants:** Mixed cancer populations (incl. lung) • **Stage:** I-IV • **Sample size:** N = 13 (Lung = 4) • **Age:** M = 58 (Range = 27-75) • **Female:** 23.07% • **Comparison:** none | Interactive voice response and SMS provide a reliable, feasible, and acceptable assessment of pain intensity and allows the rapid adaptation of treatment. Pain decreased during the intervention period. |
| Cai, 2020, USA | evaluation of framework to examine response to app-based interventions | Interventional: Single-group pilot study to test feasiblity of a framework for monitoring cancer patients | • **Participants:** Breast cancer • **Stage:** 0-III • **Sample size:** N = 7 • **Age:** M = 60 (Range = 47-75) • **Female:** 100% • **Comparison:** none | The proposed framework is deemed feasible due to high correlations between passively sensed data (i.e. location and physical activity) and mood. |
| Carson, 2021, USA | effectiveness of Mindful Yoga program RCT on daily pain and relation between daily pain and time spent in yoga practice | Interventional: randomized controlled trial | • **Participants:** Breast cancer • **Stage:** IV • **Sample size:** N = 30 • **Age:** M = 56.7 (SD = 11.7) • **Female:** 100% • **Comparison:** similar patient control group, receiving social support but no yoga intervention, with daily monitoring (N= 18) | Pain levels were low for women in both conditions, and no differential treatment effects were found for pain. A dose-response relationship was found between yoga practice duration and pain in the intervention group. A negative within-person association was found between time practicing yoga across two consecutive days and experience pain on the next day. |
| Chumbler, 2007, USA | feasibility of a Cancer Care Dialogues Model, during chemotherapy | Observational: Daily symptom reporting using home messaging device | • **Participants:** Mixed cancer populations (incl. lung) • **Stage:** I-IV • **Sample size:** N = 34 • **Age:** M = 64 (SD = 10) • **Female:** 6% • **Comparison:** none | Compliance rate was 84%. A clinical difference was detected indicating high health-related quality of life after chemotherapy. Nervousness and worry were negatively linked to health-related quality of life. |
| Çınar, 2021, Türkiye | effectiveness of mobile phone app-based training for supportive care on quality of life during adjuvant endocrine hormonal therapy | Interventional: Single-blinded, single-centered, and randomised design | • **Participants:** Breast cancer • **Stage:** I-III • **Sample size:** N = 31 • **Age:** M = 45.9 (SD = 8.3) • **Female:** 100% • **Comparison:** similar patient control group, without mobile phone app-based support (N= 33) | Quality of life of the treatment group increased after the intervention and distress level was lower compared to the control group. The mobile application was deemed informative and useful. |
| Coolbrandt, 2022, Belgium | feasibility and usability of remote system for daily symptom monitoring during persons' first systemic anticancer treatment | Observational: Descriptive research design | • **Participants:** Mixed cancer populations (incl. breast and lung) • **Stage:** 0-IIIa breast, lung not specified • **Sample size:** N = 111 (Breast = 35, Lung = 24) • **Age:** M = 59.4 (SD = 10.9) • **Female:** 51.4% • **Comparison:** none | Half of patients starting their first ever systemic treatment showed interest in using a remote system for daily symptom monitoring. Daily symptom monitoring was deemed acceptable, with a compliance rate of 59.1% during the first 12 weeks. |
| Dasch, 2010, USA | moderators of affective differentiation and daily relationship between negative and positive affect, shortly after surgery | Observational: Internet-based dairy study | • **Participants:** Breast cancer • **Stage:** 0-IV • **Sample size:** N = 53 • **Age:** M = 53.34 (SD = 9.99) • **Female:** 100% **• Comparison:** none | No within-subject moderators were found for affective differentiation. Between-subject, age and the extent of using planning to cope with cancer were positively related to affective differentiation. Whereas, anxiety, behavioral disengagement and denial coping were negatively associated with affective differentiation. |
| Dunsmore, 2023, USA | within-person fluctuations of coping and scan-related anxiety before one’s scan | Observational: Electronic daily diary study | • **Participants:** Lung cancer • **Stage:** not mentioned • **Sample size:** N = 25 • **Age:** M = 62.33 (SD = 8.10) • **Female:** 96% **• Comparison:** none | Patients reported less scan-related anxiety on days with less outcome fantasy, stagnant deliberation, and, if middle-aged, problem analysis. |
| Hachizuka, 2010, Japan | development of Personal Digital Assistant (PDA) to collect information on symptoms and mood states using computerized EMA, during home hospice care | Observational: Feasibility and usability of the device | • **Participants:** Mixed cancer populations (incl. lung) • **Stage:** IV • **Sample size:** N = 15 (Lung = 10) • **Age:** M = 67.1 (SD = 9.2) • **Female:** 20% • **Comparison:** none | The overall compliance rate to the sound of the alarm was 90.3% and 80.2% after taking rescue medications. The device was deemed user-friendly and the simplicity of content was deemed great. |
| Harper, 2012, USA | physicians' use of patients' reported daily quality of life data to evaluate their patients' response to Phase I treatment | Observational: Electronic daily diary study, usability of responses | • **Participants:** Mixed cancer populations (incl. breast and lung) • **Stage:** IV • **Sample size:** N = 30 • **Age:** M = 56.7 (SD = 12.4) • **Female:** 47% • **Comparison:** none | Physicians rated quality of life as being very important, yet, only biomedical toxicity and imaging data were used to make Phase I treatment decisions |
| Kearney, 2006, Scotland | acceptability of using handheld computer system by patients and their healthcare professionals, during two cycles of chemotherapy | Observational: Feasibility of a handheld computer system | • **Participants:** Mixed cancer populations (incl. lung) • **Stage:** not mentioned • **Sample size:** N = 15 (Lung = 8) • **Age:** M = ? (Range = 24-77) • **Female:** 33% • **Comparison:** none | Patients believed the handheld computer had improved their symptom management and felt comfortable in using it. Healthcare professionals found the handheld computer to be helpful in assessing and managing patients' symptoms. |
| Kim, 2016, South Korea | evaluation of mobile mental-health tracer, different approaches to data processing, adherence and accuracy in depression screening | Observational: evaluation of accuracy of the mobile mental-health tracker for depression screening | • **Participants:** Breast cancer • **Stage:** not mentioned • **Sample size:** N = 78 • **Age:** M = 44.35 (SD = 7.01) • **Female:** 100% • **Comparison:** none | The performance of depression screening using daily mental-health ratings gathered via mobile trackers is comparable to the results of PHQ-9. Adherence to self-reporting is associated with higher accuracy of depression screening. |
| Langer, 2018, USA | associations between enacted and perceived communication and relationship satisfaction during or after chemo- or immunotherapy, using systemic-transactional model of dyadic coping | Observational: twice-daily ecological momentary assessments to examine intra- and interpersonal associations | • **Participants:** Mixed cancer populations (incl. breast), and their partners • **Stage:** II-IV • **Sample size:** N = 107 • **Age:** M = 50.64 (SD = 12.33) • **Female:** 64.5% • **Comparison:** none | Expressing feelings was not associated with relationship satisfaction. Holding back from expressing feelings, criticizing and feeling criticized were negatively associated with relationship satisfaction. Giving and receiving support were positively associated with relationship satisfaction. |
| LeBaron, 2022,  LeBaron, 2023 (USA) | • **LeBaron et al. (2022):**  feasibility and acceptability of a smart health sensing system that was designed to support the monitoring and management of cancer pain in the home setting  • **LeBaron et al. (2023):**  descriptive summary of pain events and exploration of different types of data visualisations | Observational: smart health sensing system using ecological momentary assessments | • **Participants:** Mixed cancer populations (incl. lung), and their partners • **Stage:** III-IV • **Sample size:** N = 5 (lung = 1) • **Age:** M = ? (80% between 55-74) • **Female:** 40% • **Comparison:** none | **• LeBaron et al. (2022):**  The monitoring system is feasible and acceptable, and possibly improves patient-caregiver communication regarding pain management. Patients and caregivers mark their pain events in real-time.  **• LeBaron et al. (2023):**  Patients had an average pain severity of 5.4/10. Average caregiver-perceived distress and pain inference was higher than patient-experienced. Patients perceived caregivers to be more distressed than caregivers perceived patients to be distressed. |
| Lee, 2023, USA | reliability and validity of a subset of PRO-CTCAE items captured using a 24-hour recall | Methodological paper on observational data | • **Participants:** Mixed cancer populations (incl. breast and lung)  • **Stage:** ?-IV • **Sample size:** N = 118 (breast = 11, lung = 28) • **Age:** Median = 58 (range = 20-77) • **Female:** 40.7% • **Comparison:** none | A 24-hour recall period for PRO-CTCEA items has acceptable measurement properties and can inform on day-to-day variations in adverse events. |
| Lim & Boyle, 2022, global | investigate if food intake impacts local gastrointestinal toxicity caused by abemaciclib | Interventional: open label, randomized phase 2 trial using electronic daily diaries | • **Participants:** Breast cancer  • **Stage:** III-IV • **Sample size:** N = 72 • **Age:** M = 57.6 (73.6% < 65 years) • **Female:** 98.6% • **Comparison:** intake with meal, in fasting state, or without regard to food | Diarrhea incidence associated with abemaciclib was unrelated to timing of food intake, was predominantly low grade, of short duration and well managed. |
| Maguire, 2005, Scotland | evaluation of procedures and technical systems involved in using ASyMS for home monitoring and symptom management during chemotherapy | Interventional: pilot randomised controlled trial | • **Participants:** Mixed cancer populations (incl. breast and lung) • **Stage:** not mentioned • **Sample size:** N = 4 • **Age:** M = ? (Range = 44-74; incl. control group) • **Female:** 100% • **Comparison:** similar control group, no mobile phone monitoring (N = 6) | Patients believed that the ASyMS improved the management of their symptoms and felt reassured that they were being monitored at home. Healthcare professionals found the system beneficial in the management of symptoms and the promotion of timely interventions. |
| Maguire, 2015, UK | feasibility, acceptability and effectiveness of ASyMS during thoracic radiotherapy treatment for patients and their healthcare professionals | Observational: repeated measures, single-arm, mixed methods study design | • **Participants:** Lung cancer • **Stage:** not mentioned • **Sample size:** N = 16 • **Age:** M = 63.6 (SD = 12.9) • **Female:** 68.75% • **Comparison:** none | The handset was deemed easy to use, comprehensive and effective in supporting the management of symptoms and communication with clinicians. Patient anxiety, drowsiness and self-care efficacy improved over time. Clinicians positively perceived the system. Reducing the complexity of the system was seen as important to promote its utility. |
| McCall, 2008, Scotland | feasibility of mobile phone-based Advanced Symptom Management System in Palliative Care to monitor and manage patient symptoms at home | Observational: feasibility study to test acceptability and usability of the Advanced Symptom Management System in Palliative Care | • **Participants:** Mixed cancer populations (incl. breast and lung) • **Stage:** IV • **Sample size:** N = 21 (Breast = 3, Lung = 3) • **Age:** M = 64 (Range = 40-87) • **Female:** 33.33% • **Comparison:** none | The system was deemed usable and acceptable to patients and the health professionals who cared for them. |
| McCann, 2009;  Kearney, 2009 (UK) | • **McCann et al. (2009):** effectiveness of ASyMS on chemotherapy-related toxicity • **Kearney et al. (2009):** effectiveness of ASyMS on incidence, severity and distress of chemotherapy-related symptoms | Interventional: randomized controlled trial | • **Participants:** Mixed cancer populations (incl. breast and lung) • **Stage:** I-IV • **Sample size:** N = 56 (Breast = 34, Lung = 13) • **Age:** M = 55.1 (SD = 10.6) • **Female:** 73.2% • **Comparison:** similar patient group, with no daily monitoring (N= 56) | • **McCann et al. (2009):**  Patients reported many benefits of using ASyMS including improved communication with healthcare professionals, improvements symptom management, and feeling reassured their symptoms were being monitored at home.  • **Kearney et al. (2009):**  There were lower reports of fatigue and higher reports of hand-foot syndrome in the intervention group compared to the control group. |
| Min, 2014, South Korea | feasibility of an app for sleep disturbance-related data collection during neoadjuvant chemotherapy and evaluation of factors related to better compliance | Observational: feasibility of using a smartphone app | • **Participants:** Breast cancer • **Stage:** I-III • **Sample size:** N = 30 • **Age:** M = 45.6 (SD = 6) • **Female:** 100% • **Comparison:** none | Overall compliance of assessments was 45% over 90 days, with a median of 41.1% (range 6.7-95.6%). Compliance decreased over time. Compliance was higher for unemployed women and women who started to self-report on the day right after enrollment. |
| Mooney, 2014, USA | effectiveness of email-alert reports to clinicians when symptom exceeded moderate-to-severe intensity level threshold during two cycles of ambulatory chemotherapy | Interventional: randomized controlled trial | • **Participants:** Mixed cancer populations (incl. breast and possibly lung) • **Stage:** I-IV • **Sample size:** N = 129 • **Age:** M = 55.2 (Range = 21-86) • **Female:** 82.2% • **Comparison:** similar patient group, with daily monitoring but no clinical action (N= 121) | Patients reported high satisfaction with and ease of use of the system. Over 80% of providers reported usefulness of symptom alert reports. An average of nine moderate-to-severe intensity alerts were generated per patient. However, providers rarely contacted patients after receiving alerts. Symptom severity did not change differently between groups. |
| Nordhausen, 2022, Germany | plan, conduct, and evaluate implementation of electronic patient-reported outcomes assessment in an inpatient radiation oncology clinic | Observational: single-center implementation study | • **Participants:** Mixed cancer populations (incl. breast and lung) • **Stage:** 68% at least one secondary site • **Sample size:** N = 344 (breast = 6, lung = 106) • **Age:** M = 63.7 (SD = 11.0) • **Female:** 37.2% • **Comparison:** none | An assessment system was developed with baseline, daily, and discharge questionnaires using EORTC items. The system is acceptable for patients and identified more symptoms followed by more supportive measures compared to not using the system. |
| Passardi, 2022, Italy | customize ONCO-TreC, evaluate its ability to facilitate shared management of oral anticancer therapies and evaluate its usability and acceptability | Interventional: Prospective training-validation, interventional, nonpharmacological, multicenter study | • **Participants:** Mixed cancer populations (incl. breast and lung) • **Stage:** ?-IV • **Sample size:** N = 40 • **Age:** Median = 66.42 (Range = 42-82) • **Female:** 60% • **Comparison:** none | The system is effective in measuring adherence to treatment. Patients reported less adverse events in the app than physicians in case report forms, especially when events were less severe. Most patients opened the app once a week and used the app for messages and vital sign entering at least once. |
| Pinto, 2021, USA | longitudinal trends in sedentary behavior and associated variables | Observational study | • **Participants:** Breast cancer • **Stage:** 0-III • **Sample size:** N = 22 • **Age:** M = 51.5 (SD = 8.4) • **Female:** 100% • **Comparison:** none | Baseline sedentary behavior averaged 76.7 hours per week. Sadness, anxiety, stress, worry, and fatigue were positively associated with sedentary behavior while positive affect was negatively associated with sedentary behavior. Being at work was associated with more sedentary behavior. Cross-lagged effects were found for affective valence, fatigue, and sedentary behavior. |
| Ratcliff, 2014, USA | relation of sleep before and during chemotherapy treatment and symptoms and mood during chemotherapy treatment | Observational: self-report measures and EMA to assess temporal associations | • **Participants:** Breast cancer • **Stage:** I-III • **Sample size:** N = 20 • **Age:** M = 54.70 (SD = 10.29) • **Female:** 100% • **Comparison:** none | Sleep disturbance prior to chemotherapy infusion was positively associated with fatigue, and negative and anxious mood throughout the 3-week chemotherapy cycle. Good sleep before infusion buffered anxious mood in the first days following infusion. Sleep latency and fragmentation had different effects on symptoms and mood during the day. |
| Schuler, 2023, Australia | feasibility of wearable sensor-triggered ecological momentary assessments and electronic patient-reported outcomes in community palliative care | Observational: ecological momentary assessments and weekly electronic patient-reported outcome surveys | • **Participants:** Mixed cancer populations (incl. lung and possibly breast), and their partners • **Stage:** III-IV • **Sample size:** N = 15 (lung = 2; 30 dyads) • **Age:** M = 59 (range = 35-78) • **Female:** 80% • **Comparison:** none | Participants adhered to wearing the smartwatches and perceived value in this support. Quantity and severity of ‘‘stress’’ events were higher in patients. Sleep disturbance was similar but for different reasons: patients (physical symptoms) and caregivers (worrying about the patient). |
| Shiyko, 2014; Shiyko, 2019 (USA) | • **Shiyko et al. (2014):**  introduction of generalized time-varying effect model, as semi-parametric approach for investigating time-varying effects of a treatment • **Shiyko et al. (2019):** natural capacity to exhibit mindfulness states during recovery from surgery | • **Shiyko et al. (2014):**  Methodological paper on observational data • **Shiyko et al. (2019):**  Observational: prospective, longitudinal, non-randomised study | • **Participants:** Lung cancer (N-SC), undergoing traditional open thoracotomy and lobectomy or undergoing minimally invasive video-assisted variant • **Stage:** I • **Sample size:** N = 59 (36 in video-assisted surgery group) • **Age:** M = 66.1 (SD = 12.9) • **Female:** 61% • **Comparison:** none | • **Shiyko et al. (2014):**  The introduced model identified a pattern of an effect that varied in time and magnitude, where traditional analyses did not. Group differences manifested after day four. • **Shiyko et al. (2019):** For cancer patients recovering from surgery, the innate ability to exhibit mindfulness is limited. |
| Solk, 2019; Phillips, 2020;  Auster-Gussman, 2021;  Welch, 2023;  Whitaker, 2023 (USA) | • **Solk et al. (2019):** feasibility and acceptability of using EMA to assess activity, symptoms, and motivation during chemotherapy cycle • **Phillips et al. (2020):** relation between daily symptoms and activity during chemotherapy • **Auster-Gussman et al. (2021):**  relation between social cognitive theory constructs and physical activity during chemotherapy  • **Welch et al. (2023):**  associations between daily fluctuations in symptoms and sedentary behavior during chemotherapy  • **Whitaker et al. (2023):**  association between objective physical activity and symptoms during chemotherapy | Observational: prospective longitudinal study using ecological momentary assessments and passive physical activity measures | • **Participants:** Breast cancer • **Stage:** I-III • **Sample size:** N = 67-68 • **Age:** M = 48.6 (SD = 10.3) • **Female:** 100% • **Comparison:** none | • **Solk et al. (2019):** Compliance rate was 84.14% and valid accelerometer data was gathered on 82% days. All patients were confident in their ability to use study technology and the majority had a positive study experience. Reactivity varied, with more than half of patients indicating that the study methods made them want to increase activity. • **Phillips et al. (2020):** Within-person associations were found between physical activity and same day affect, fatigue, pain, walking, activities of daily living physical function, and cognitive function. Previous-day anxiety was associated with next-day light physical activity. • **Auster-Gussman et al. (2021):**  Within-person changes in self-efficacy, physical outcome expectations and day goal setting were positively related with physical activity on the same day.  • **Welch et al. (2023):**  Higher symptom ratings were associated with increased sedentary behaviors later in time and higher sedentary behaviors were associated with worse later symptoms  • **Whitaker et al. (2023):**  Within person higher than usual positive affect on a given day, regardless of intensity, is associated with improved symptom ratings on the current and next day. |
| Steffen, 2018;  Steffen, 2020 (USA) | • **Steffen et al. (2018):**  relation of daily hope and daily stigma with same- and next-day functioning during cancer treatment • **Steffen et al. (2020):** associations among aspects of hope agency and pathways thinking, daily fatigue, pain and functional concerns during active treatment | Observational: daily assessment methodology | • **Participants:** Lung cancer • **Stage:** IIIa-IV (N-SC) and I-IV (SC) • **Sample size:** N = 50 • **Age:** M = 68.7 (SD = 8.8) • **Female:** 58% • **Comparison:** none | • **Steffen et al. (2018):**  Hope was positively associated with same- and next-day functioning. The effect of treatment days on role and social functioning was moderated by hope. Within-person hope was not predictable by disease symptoms. Within-person, stigma was negatively related to functioning. • **Steffen et al. (2020):** Within- and between-person associations were found between increased pathways and agency thinking and lower symptoms and better functioning. |
| Stone, 2016, USA | ecological validity of eight retrospective PROMIS measurement systems by comparing with daily diary data gathered during chemotherapy | Observational: daily diary methodology | • **Participants:** Breast cancer • **Stage:** I-III • **Sample size:** N = 86 • **Age:** M = 51.2 (SD = 9.7) • **Female:** 100% • **Comparison:** Community residents (N = 98), osteoarthritis patients (N = 98), women experiencing premenstrual syndrome (N = 93), men undergoing hernia surgery (N = 98) | Non-diary PROMIS scales replicated between-group differences in diaries and week-to-week changes in diaries. Diaries were correlated to non-diary PROMIS scale. |
| Sztachańska, 2019, Poland | effectiveness of gratitude intervention | Interventional: effectiveness of a gratitude intervention (and within-person relationships between gratitude and daily psychological functioning). | • **Participants:** Breast cancer • **Stage:** not mentioned • **Sample size:** N = 21 • **Age:** M = 46.86 (SD = 9.04) • **Female:** 100% • **Comparison:** similar control group, identical monitoring without reporting reasons why grateful (N = 21) | Listing reasons for gratitude led to higher levels of daily psychological functioning, greater perceived support, and greater use of adaptive coping strategies. |
| van den Berg, 2022, USA | feasibility and accuracy of daily micro-surveys before and after surgery | Observational: prospective study evaluating the feasibility and descriptive quality of capturing PROMs through daily micro-surveys | • **Participants:** Mixed cancer populations (incl. breast) • **Stage:** not mentioned • **Sample size:** N = 95 (Breast = 31) • **Age:** M = 52.1 (SD = 12.9) • **Female:** 66% • **Comparison:** none | Compliance rates for the full-length SF-36 and micro-surveys were 76% and 34%. More SF-36 surveys were collected using the daily micro-surveys compared to the intermittent full-length SF-36. A lack of agreement was found between micro-surveys and SF-36, which improved with higher micro-survey completion rate. The daily micro-surveys were deemed as not burdensome. |
| van Roozendaal, 2023, the Netherlands | dynamic associations among fatigue, and its perpetuating and protective factors | Observational: ecological momentary assessment study | • **Participants:** Mixed cancer populations (incl. breast and possibly lung) • **Stage:** I-IV • **Sample size:** N = 30 (Breast = 15) • **Age:** M = 50.4 (SD = 9.7) • **Female:** 76.7% • **Comparison:** none | Concurrently, higher acceptance and self-efficacy were associated with lower fatigue, whereas all other factors were related with higher fatigue. Higher self-efficacy preceded lower fatigue in time, whereas higher fatigue preceded increased allowing rest. |
| Weaver, 2014, England | relative dose intensity of capecitabine, level of toxicity and perceived supportive care | Interventional: pilot, one arm, single-center clinical study | • **Participants:** Mixed cancer populations (incl. breast) • **Stage:** IV • **Sample size:** N = 26 (Breast = 8) • **Age:** M = 53 (Range = 37-66) • **Female:** 100% • **Comparison:** none | Compliance rate was 92.6%. The maximum toxicity grade was reached by 46.1% of patients. The average dose intensity relative to standard dose was 90%. The dose was changed in more than half of the patients. The monitoring system was perceived as reassuring, by both patients and healthcare professionals. |
| Xu, 2019, China | association of illness representation and fear of cancer recurrence in daily life in couples, considering daily couple communication | Observational: daily momentary assessment study | • **Participants:** Breast cancer, and their partners • **Stage:** I-III • **Sample size:** N = 54 • **Age:** M = 44.6 (SD = 8.39) • **Female:** 100% • **Comparison:** none | Between‐person, more severe illness representation was related to more negative and less positive information expression in daily life. Within-person, perceptions of positive information acted as mediator between information disclosures and fear of cancer recurrence. |
| Yap, 2013, Singapore | feasibility and acceptability of pharmacist-ran tele-oncology service to monitor chemotherapy-induced nausea and vomiting in outpatients | Observational: single-center prospective study to assess feasibility of a pharmacist-run tele-oncology service using SMS | • **Participants:** Mixed cancer populations (incl. breast and lung) • **Stage:** I-IV • **Sample size:** N = 60 (Breast = 19, Lung = 3) • **Age:** Median = 49.5 (IQR = 42.3-55.0) • **Female:** 65% • **Comparison:** none | Seventy-three percent of patients completed all assessments. The majority were comfortable with the duration of SMS monitoring, especially adherent patients. Over half found the SMS advice useful. The pharmacists made 22 calls for uncontrolled chemotherapy-induced nausea and vomiting. |

**Abbreviations.** ASyMS = Advanced Symptom Management System; EMA; Ecological Momentary Assessments; PDA = Personal Digital Assistant; PRO-CTCAE = patient-reported outcome version of the common terminology criteria for adverse events; SMS = Short Messaging System
