# Supplementary material for: Intensive Longitudinal Methods Among Adults With Breast or Lung Cancer: Scoping Review
Source: J Med Internet Res. 2024 Jun 12;26:e50224. doi: 10.2196/50224 (PMC11208836; doi:10.2196/50224)
Supplement: Multimedia Appendix 3 [file jmir_v26i1e50224_app3.docx]

**Multimedia Appendix 3.** Content and design characteristics

| **Study** | **Questionnaire length (in number of items)** | **Constructs measured daily (measurement instrument or source)** | **System characteristics** | **Sampling schedule characteristics** | **Supportive features** |
| --- | --- | --- | --- | --- | --- |
| Aigner et al. (2016) | 6 | • intensity of pain throughout day  • sad and anxious mood • urge to smoke • cigarette use • usage of over-the-counter and prescription pain medication | • **device (operating system):** HP iPAQ H1945 Palmtop personal computer (Window Mobile 5);  • **application:** software program developed specifically for ecological momentary assessment | • **monitoring periods:** 1 • **period duration:** 14 days • **daily prompt frequency:** 1 • **sampling type:** fixed signal-contingent or interval-contingent • **prompt interval:** unclear if prompt used | none mentioned |
| Badr et al. (2010); Badr et al. (2013); Stephenson et al. (2018) (reporting on the same study) | 13 - 14 (depending on responses) | • **Badr et al. (2010):  *repeated measurements during day:***   • patient pain severity (BPI)  • analgesic use and pain relief (adapted from BPI)  • mood (circumplex adjectives, identified by Larsen and Diener, 1992) ***once in the evening:***  • provision/receipt of social support  • degree to which cancer interfered with their relationship  • **Badr et al. (2013):  *once in the morning:***  • mood (circumplex adjectives, identified by Larsen and Diener, 1992) ***once in the evening:***  • avoidance of discussing cancer-related concerns with partner that day   • **Stephenson et al. (2018): *repeated measurements during day:***   • momentary pain (BPI)  • pain medication use (adapted from BPI) | • **device (operating system):** Palm Tungsten E or E2 computers (32MB RAM) with stylus • **application:** not mentioned | • **monitoring periods:** 1 • **period duration:** 14 days • **daily prompt frequency:** 6 • **sampling type:** time-blocked random signal-contingent  • **prompt interval:** at least 30 minutes | none mentioned |
| Belcher et al. (2011); Pasipanodya et al. (2012) (reporting on the same study) | 52 - approximately 84 (depending on responses) | • **Belcher et al. (2011):**  • relationship happiness   • momentary relationship intimacy (based on Laurenceau, Feldman-Barrett, & Pietromonaco, 1998)  • momentary negative affect  • support provision  • support receipt (based on Bolger et al., 2000) • **Pasipanodya et al. (2012):**  • relationship happiness (Dyadic Adjustment Scale)  • momentary relationship intimacy (based on Laurenceau, Feldman-Barrett, & Pietromonaco, 1998)  • momentary positive and negative affect (PANAS-X)   • momentary self-esteem (Self Esteem Scale)  • daily events and sharing them with partner | • **device (operating system):** patient-owned device with internet access • **application:** secure server browser-based survey | • **monitoring periods:** 1 • **period duration:** 7 days • **daily prompt frequency:** 1 • **sampling type:** fixed signal-contingent  • **prompt interval:** 1 day | none mentioned |
| Otto et al. (2015) - combination of two datasets, including Belcher et al. (2011) | 52 - approximately 84 (depending on responses) | • daily capitalization and social support attempts for positive and negative events respectively (used in Pasipanodya et al., 2012) • event-related daily perceived partner responsiveness • momentary ratings of positive and negative affect (PANAS-X) • momentary intimacy (Dyadic Adjustment Scale) | • **device (operating system):** patient-owned device with internet; not mentioned • **application:** secure server browser-based survey; not mentioned | • **monitoring periods:** 1 • **period duration:** 7 or 10 days (depending on study) • **daily prompt frequency:** 1 • **sampling type:** fixed signal-contingent  • **prompt interval:** 1 day | none mentioned |
| Besse et al. (2016) | 1 | • pain severity (Numeric Rating Scale) | • **device (operating system):** patient-owned mobile phone device • **application:** SMS and interactive voice response system | • **monitoring periods:** 1 • **period duration:** 16 days • **daily prompt frequency:** 2 • **sampling type:** fixed signal-contingent  • **prompt interval:** at least 5 hours and 30 minutes | • **clinician alerts:** if pain was moderate or high, the nurse contacted the patient and, if required, adapted the treatment |
| Cai et al. (2020) | at least 5 | • mood • anxiety • depression • social functioning • health-related behavior | • **device (operating system):** patient-owned smartphone device • **application:** SMS tool by Qualtrics | • **monitoring periods:** 1 • **period duration:** 7 days • **daily prompt frequency:** 1 • **sampling type:** fixed signal-contingent  • **prompt interval:** 1 day | • **clinician informing** (possibly)**:** coaching after diary period (not mentioned if diary data is used during coaching session) |
| Carson et al. (2021) | 5 | • pain (used in Carson et al., 2009) • in yoga condition: time spent in yoga, meditation and breathing practice | • **device (operating system):** patient-owned telephone device • **application:** interactive telephone voice system (toll-free number to call) | • **monitoring periods:** 4 • **period duration:** 7 days • **daily prompt frequency:** 1 • **sampling type:** not mentioned • **prompt interval:** unclear if prompt used | none mentioned |
| Chumbler et al. (2007) | not specified, at least 5 | • pain (adapted from MD Anderson Symptom Inventory) • fatigue (adapted from MD Anderson Symptom Inventory) • functional limitations (adapted from MD Anderson Symptom Inventory) • nausea (adapted from MD Anderson Symptom Inventory) • nervousness/worry (adapted from MD Anderson Symptom Inventory) | • **device (operating system):** Health Buddy appliance (by Health Hero Network) • **application:** device-specific software | • **monitoring periods:** 1 • **period duration:** between 30 and 180 days (*M* = 120) • **daily prompt frequency:** 1 • **sampling type:** not mentioned • **prompt interval:** unclear if prompt used | • **automated self-care advice:** relevant to the state of each symptom |
| Çınar et al. (2021) | at least 3 questions per experienced symptom | **•** experienced symptoms (open question) • symptom severity  • situations that increase or improve severity • (Picture in article shows open question for "treatment" and "medication") | • **device (operating system):** patient-owned smartphone (Android) • **application:** application developed by the study authors | • **monitoring periods:** 1 • **period duration:** 84 days • **daily prompt frequency:** 1 • **sampling type:** daily reminder to regularly record symptom diary • **prompt interval:** 1 day | **app consisted of the following modules:**  • training (information on breast cancer, treatment methods, problems and suggestions in endocrine hormonal therapy),  • symptom diary,  • questions (communication with research specialist nurse),  • reminders (daily diary reminder, practice relaxation techniques, etc.),  • opinions (after study) |
| Coolbrandt et al. (2022) | not specified, presumably 11 - 21 (depending on responses) | **•** basic set of symptoms: nausea, vomiting, anorexia, diarrhea, constipation, fatigue, pain, rash, dyspnea, psychosocial burden, fever (paper version of diary was already in use; Coolbrandt et al., 2017) • optional treatment-tailored symptoms: oral mucositis, peripheral neuropathy, tearing eyes, hearing loss, hand-foot syndrome, itch, myalgia and arthralgia, cough, and hypertension, intake of oral anticancer medication (paper version of diary was already in use; Coolbrandt et al., 2017) | • **device (operating system):** patient-owned smartphone (Android or iOS), pen-and-paper possible (166 of 465 choose paper, 163 mobile, 73 web version) • **application:** Mynexuzhealth application | • **monitoring periods:** 1 • **period duration:** up to 84 days • **daily prompt frequency:** 1 • **sampling type:** daily reminder to record symptom diary • **prompt interval:** 1 day | • **automated self-care advice:** with possibility to read more on hospital's website. In case of severe symptoms, feedback urges to contact healthcare professional.  • **clinician alerts:** severe symptoms alert hospital clinician to contact patient  • **clinician informing:** summary and all completion visible in the patient file |
| Dasch et al. (2010) | 40 | • momentary negative and positive affect (PANAS-X) • daily positive and negative events (based on consultation with hospital staff and existing daily event checklists) | • **device (operating system):** patient-owned device with internet access • **application:** browser-based daily diary | • **monitoring periods:** 1 • **period duration:** 7 days • **daily prompt frequency:** 1 • **sampling type:** interval-contingent (between 7pm and 11pm) • **prompt interval:** no prompt | none mentioned |
| Dunsmore & Neupert (2023) | 21 | • anticipatory coping (adapted from Measure of Mental Anticipatory Processes) • scan-related anxiety (modified IES-6) | • **device (operating system):** presumably patient-owned device  • **application:** survey sent via email | • **monitoring periods:** 1 • **period duration:** 7 days • **daily prompt frequency:** 1 • **sampling type:** signal/interval-contingent (email sent at 6am) • **prompt interval:** not mentioned | none mentioned |
| Hachizuka et al. (2010) | 6 | • pain • fatigue • nausea • anxiety • depression • drowsiness | • **device (operating system):** Sharp Zaurus Model SL-C1000 handheld PDA • **application:** not mentioned | • **monitoring periods:** 1 • **period duration:** 7 days • **daily prompt frequency:** 3 to 5 • **sampling type:** time-blocked random and fixed signal- and event-contingent whenever rescue medications were taken • **prompt interval:** not mentioned | none mentioned |
| Harper et al. (2012) | 15 | **•** daily quality of life (EORTC QLQ-C15-PAL) | • **device (operating system):** research-provided portable handheld device • **application:** not mentioned | • **monitoring periods:** 1 • **period duration:** between 4 and 142 days (*M* = 52) • **daily prompt frequency:** 1 • **sampling type:** not mentioned • **prompt interval:** unclear if prompt used | • **clinician informing:** physicians were provided with a summary of patients' quality of life reports before each visit (lowest, highest and average quality of life of past 7 days) |
| Kearney et al. (2006) | not specified, presumably 6 | **•** fatigue (adapted from Chemotherapy Symptom Assessment Scale) • nausea and vomiting (adapted from Chemotherapy Symptom Assessment Scale) • oral problems (adapted from Chemotherapy Symptom Assessment Scale) • diarrhea (adapted from Chemotherapy Symptom Assessment Scale) • breathlessness (depending on diagnosis; adapted from Chemotherapy Symptom Assessment Scale) | • **device (operating system):** research-provided handheld computer • **application:** handheld computer system created through intensive design and feasibility work in collaboration with patients | • **monitoring periods:** 1 • **period duration:** 7 days • **daily prompt frequency:** 1 • **sampling type:** not mentioned • **prompt interval:** not mentioned | • **automated self-care advice:** specific to symptom reports.  • **clinician alerts:** nurse contacted patients if patient had certain score and provided further symptom management advice.  • **clinician informing:** daily symptom reports were stored in the patient's cases notes for review by the healthcare team prior to the patient's next chemotherapy cycle. |
| Kim et al. (2016) | 3 | • anxiety • mood • sleep satisfaction | • **device (operating system):** not mentioned • **application:** Pit-a-Pat | • **monitoring periods:** 1 • **period duration:** up to 336 days • **daily prompt frequency:** presumably 1 • **sampling type:** not mentioned • **prompt interval:** unclear if and how frequently prompt used | none mentioned |
| Langer et al. (2018) | 10 - 11 (depending on answers) | ***repeated measurements during day:*   •** conversations related to cancer  • expression of and holding back from expression (adapted from Emotional Disclosure Scale)  • support and criticism of partner  • parallel ratings of partner behavior ***once in the evening:***  • relationship satisfaction (Dyadic Adjustment Scale) | • **device (operating system):** patient-owned smartphone device (n = 92) or research-provided iPod Touch (n = 15) • **application:** lifedatacorp.com | • **monitoring periods:** 1 • **period duration:** 14 days • **daily prompt frequency:** 2 • **sampling type:** fixed signal-contingent • **prompt interval:** 8 hours | none mentioned |
| LeBaron et al. (2022);  LeBaron et al. (2023)  (reporting on the same study) | • **pain events:** 7 • **daily prompt:** 12 | ***Pain events during the day:*   •** pain severity  **•** (partner) distress  **•** opioid use  ***once per day:***  • pain, distress, and other factors influencing pain (i.e., sleep quality, mood, activeness, and interactions) | • **device (operating system):** researcher-provided Fossil Sport Gen 4 smartwatches (BESI-C WearOS) • **application:** part of BESI-C system | • **monitoring periods:** 1 • **period duration:** 10-14 days • **daily prompt frequency:** at least 1 • **sampling type:** event-contingent when pain (and signal-contingent 30 minutes later), and signal-contingent once daily • **prompt interval:** 24 hours (between daily assessments) | none mentioned |
| Lee et al. (2023) | 27 | Symptomatic adverse events (24h recall version of PRO-CTCAE): anxiety, sad or unhappy feelings, constipation, loose stools, loss of appetite, nausea, shortness of breath, numbness or tingling in hands and feet, pain, fatigue, insomnia, dry mouth, mouth or throat sores, and vomiting | • **device (operating system):** patient-owned telephones • **application:** automated telephone interactive voice response | • **monitoring periods:** 1 • **period duration:** 28 days • **daily prompt frequency:** 1 • **sampling type:** not mentioned • **prompt interval:** unclear if prompt used | none mentioned |
| Lim et al. (2022) | not specified, presumably 5 | • number of bowel movements and diarrhea  • intake of loperamide  • intake of treatment in relation to meal | • **device (operating system):** not mentioned • **application:** not mentioned | • **monitoring periods:** 1 • **period duration:** 84 days • **daily prompt frequency:** not mentioned • **sampling type:** not mentioned • **prompt interval:** unclear if prompt used | none mentioned |
| Maguire et al. (2005) | not specified, presumably 6 | • incidence, severity and distress common chemotherapy related symptoms: nausea, vomiting, mucositis, hand-foot syndrome, diarrhea and fatigue (selected from Common Toxicity Criteria Adverse Events grading system and the Chemotherapy Symptom Assessment Scale) • body temperature | • **device (operating system):** mobile phone (unclear if provided by researchers or patient-owned) • **application:** ASyMS developed over period of 5 years | • **monitoring periods:** 1 • **period duration:** 14 days • **daily prompt frequency:** 2 • **sampling type:** signal- or interval-contingent, and event-contingent whenever feeling unwell • **prompt interval:** unclear if prompt used | • **automated self-care advice** • **clinician alerts:** development of a risk model which alerted healthcare providers of symptoms that were severe or life-threatening |
| Maguire et al. (2015) | unclear | • physical symptoms, psychological symptoms, distress (Memorial Symptom Assessment Scale - Short Form) • ability to do activities (Rotterdam Symptom Checklist - Activity Subscale) | • **device (operating system):** mobile phone (unclear if provided by researchers or patient-owned) • **application:** ASyMS | • **monitoring periods:** 2 • **period duration:** 7 days of chemotherapy and 30 days post-treatment • **daily prompt frequency:** not mentioned • **sampling type:** not mentioned • **prompt interval:** not mentioned | • **automated self-care advice** • **clinician alerts:** based on symptom reports. amber alert in case early interventions were advised, and for red alerts the clinicians contacted the patient as soon as possible. |
| McCall et al. (2008) | unclear | • incidence, severity and distress of common palliative symptoms:   - nausea, vomiting, fatigue, constipation/diarrhea (adapted from Chemotherapy Symptom Assessment Scale)  - pain (adapted from Brief Pain Inventory Short Form)  - distress (adapted from National Comprehensive Cancer Network Distress Management Thermometer) | • **device (operating system):** personal digital assistant  • **application:** not mentioned | • **monitoring periods:** 1 • **period duration:** 30 days • **daily prompt frequency:** 1 • **sampling type:** interval-contingent and event-contingent whenever feeling unwell • **prompt interval:** no prompt | • **automated self-care advice** • **insight into responses:** symptom graphs |
| McCann et al. (2009);  Kearney et al. (2009) (reporting on the same study) | not specified, presumably 7 | • **McCann et al. (2009):**  • incidence, severity and distress of nausea, vomiting, mucositis, hand-foot syndrome, diarrhea and fatigue  • body temperature  • **Kearney et al. (2009):**   • incidence, severity and distress of nausea, vomiting, mucositis, hand-foot syndrome, diarrhea and fatigue (selected from CTCAE grading system and the Chemotherapy Symptom Assessment Scale) | • **device (operating system):** mobile phone (unclear if provided by researchers or patient-owned) • **application:**  ASyMS developed over period of 5 years | • **monitoring periods:** 4 • **period duration:** 14 days • **daily prompt frequency:** 2 • **sampling type:** signal- or interval-contingent, and event-contingent whenever feeling unwell • **prompt interval:** unclear if prompts used | • **automated self-care advice** • **clinician alerts:** based on symptom reports (amber alert in case early interventions were advised, and for red alerts the clinicians contacted the patient as soon as possible, usually within an hour). |
| Min et al. (2014) | 6 | ***once daily:***   • sleep-disturbance symptoms related to mild depression, including anxiety and mood ***available in app, but unclear if used in study:***  • acute symptoms related to chemotherapy  • medication diary for antihormonal treatment | • **device (operating system):** patient-owned smartphone  • **application:** Pit-a-Pat | • **monitoring periods:** 1 • **period duration:** 90 days • **daily prompt frequency:** twice daily, but only one daily assessment to complete • **sampling type:** fixed signal-contingent • **prompt interval:** at least 9 hours | • **clinicians informing**: clinicians reviewed at 3-week intervals the self-reported data (however, as part of the study conclusion, clinicians did not differ in the way they managed reported symptoms from reviewed patients and non-reviews patients) |
| Mooney et al. (2014) | 11-31 (depending on answers) | • presence, severity and distress of pain, fatigue, nausea/vomiting, fever, trouble sleeping, anxiety, depressed mood, sore mouth, diarrhea, and constipation • occurrence of vomiting episodes, oral intake, dizziness, use of anti-emetics for nausea, and physician contacts | • **device (operating system):** patient-owned telephone device • **application:** telephone automated monitoring system (touch-tone keypad) | • **monitoring periods:** 1 • **period duration:** *M* = 45 days • **daily prompt frequency:** 1 • **sampling type:** signal- or interval-contingent • **prompt interval:** unclear if prompts used | • **clinician alerts:** oncologist and oncology nurse received immediate symptom alert reports through fax or mail (including answers and graphs), threshold based on severity or distress level and trend over days (based on pilot) |
| Nordhausen et al. (2022) | 14-19 (depending on diagnosis) | • core symptoms (EORTC Item Library)  • specific symptoms of most frequently treated cancer sites in the clinic | • **device (operating system):** hospital-provided tablets or bedside devices • **application:** Computer-based Health Evaluation System | • **monitoring periods:** 1 • **period duration:** 1-36 days • **daily prompt frequency:** presumably 1 • **sampling type:** “patient contacts” • **prompt interval:** not mentioned | • responses monitored by hospital staff to improve staff-patient communication and targeted supportive measures |
| Passardi et al. (2022) | not mentioned | • adverse events (previously developed application by Passardi et al., 2017) • number of pills (previously developed application by Passardi et al., 2017) • vital signs (previously developed application by Passardi et al., 2017) | • **device (operating system):** not mentioned (presumably smartphone) • **application:** ONCO-TreC | • **monitoring periods:** ? • **period duration:** *M* = 4.4 months (*SD* = 8.0) • **daily prompt frequency:** not specified • **sampling type:** fixed signal-contingent for vital signs, sampling type for other diary module not mentioned • **prompt interval:** unclear if and how many prompts used | • **information:** visual reminder of cancer therapy, a reminder of concomitant drugs to be taken and messaging system  • **communication:** software allowed patients to contact health professionals |
| Pinto et al. (2021) | morning: at least 22 later during day: 23 | • activities (excluding the first assessment of the day) • affect (general, sadness, anxiety, stress; based on previous study by Hardy and Rejeski, 1989) • symptoms (neuropathy, lymphedema, pain, fatigue and illness) • worry about cancer | • **device (operating system):** patient-owned smartphone (Android or iOS) • **application:** mobile EMA ilumivu app | • **monitoring periods:** 5 • **period duration:** 7 days • **daily prompt frequency:** 5 • **sampling type:** random signal-contingent • **prompt interval:** at least 3 hours | none mentioned |
| Ratcliff et al. (2014) | morning: 5 later during day: 20 | ***once in the morning:***   • sleep (minutes spent in bed, sleep latency, sleep fragmentation, fragmentation durations, overall quality)  ***repeated assessments during day:***   • mood (circumplex model of emotion measure adjectives, same adjective only once per day and different pairings)  • symptom severity (nausea, fatigue, difficulty concentrating, and numbness; based on focus groups with 50 breast cancer patients) | • **device (operating system):** Casio E-100 PPC (Windows CE) • **application:** custom-programmed software system | • **monitoring periods:** 5 • **period duration:** until next chemotherapy visit (*M* = 20.15 days, *SD* = 1.84) • **daily prompt frequency:** 4 • **sampling type:** time-blocked random signal-contingent • **prompt interval:** at least 2 hours | none mentioned |
| Schuler et al. (2023) | 3 | ***Stress-triggered questionnaire:***  • Distress  ***Once every morning:***  • Sleep | • **device (operating system):**  Garmin vivosmart 4 smartwatches & patient-owned smartphones  • **application:** Garmin Connect & ilumivu mEMA | • **monitoring periods:** 1 • **period duration:** 33 • **daily prompt frequency:** at least one • **sampling type:** fixed signal-contingent and event-contingent triggered by passively measured stress • **prompt interval:** 24 hours (between daily assessments) | none mentioned |
| Shiyko et al. (2014);  Shiyko et al. (2019) (reporting on the same study) | 11-18 (depending on anwers) | • **Shiyko et al. (2014):**  • frequency and burden of physical symptoms (pain, energy level, drowsiness, appetite intensity, constipation, shortness of breath, and chest tightness; Memorial Symptom Assessment Scale)  • **Shiyko et al. (2019):**  • mindfulness (adapted from earlier version of Toronto Mindfulness Scale) | • **device (operating system):** research-provided portable palm pilot • **application:** not mentioned | • **monitoring periods:** 1 • **period duration:** 14 days • **daily prompt frequency:** 2 • **sampling type:** time-blocked random signal-contingent • **prompt interval:** not mentioned | none mentioned |
| Solk et al. (2019); Phillips et al. (2020); Auster-Gussman et al. (2021);  Welch et al. (2023); Whitaker et al. (2023) (reporting on the same study) | 7-13 (depending on prompt timing) | • **Solk et al. (2019):** ***repeated assessments during day:***   • symptom burden (affect, depression, anxiety, fatigue, pain, physical and cognitive function; adapted from PROMIS)  • motivational factors for increasing physical activity and reducing sedentary behavior (morning and evening; based on literature on self-efficacy, outcome expectations and goal-setting) ***once in the morning:***  • sleep quality (adapted from PROMIS) ***once in the evening:***  • minutes and level of exercise (modified Godin Leisure Time Exercise Questionnaire)  • **Phillips et al. (2020):** ***repeated assessments during day:***   • affect (based on scale of Hardy et al., 1989)  • anxiety (adapted from PROMIS)  • depression (adapted from PROMIS)  • fatigue (adapted from PROMIS)  • physical function (adapted from PROMIS)  • pain (adapted from PROMIS)  • cognitive function (adapted from PROMIS)  • **Auster-Gussman et al. (2021): *morning and evening assessments:***   • same-day and next-day exercise self-efficacy (Yost et al., 2011)  • physical and psychological outcome expectations (Wojcicki et al., 2009)  • goal-setting (McAuley et al., 1993)  • **Welch et al. (2023):**  • symptom burden (modified PROMIS items): affect, anxiety, depression, fatigue, physical functioning, pain, and cognitive functioning  • sedentary behaviors  • **Whitaker et al. (2023):**  • symptom burden analogous to Welch et al. (2023)  • physical activity | • **device (operating system):** patient-owned smartphone • **application:** text-messages directed to browser-based survey | • **monitoring periods:** 3 • **period duration:** 10 days • **daily prompt frequency:** 4 • **sampling type:** random signal-contingent • **prompt interval:** at least 2 hours | none mentioned |
| Steffen et al. (2018);  Steffen et al. (2020) (reporting on the same study) | 28 | • **Steffen et al. (2018):**  • momentary affect (adapted from PANAS-X)  • momentary hope (adapted from State Hope Scale)  • lung cancer stigma (Cataldo Lung Cancer Stigma Scale)  • physical symptoms (adapted from FACT-L)  • treatment factors   • social/role and physical functioning (adapted from EORTC-QLQ-C30) **• Steffen et al. (2020):**  • symptom cluster (fatigue and pain; based on Given et al., 2001)   • functional concern (feeling useful, independence, making each day count, feeling like a burden to family; adapted from FACIT-Pal) | • **device (operating system):** online version (chosen by 38%), paper (56%), or telephone call with interviewer (6%) • **application:** email direct to browser-based survey (or paper version) | • **monitoring periods:** 1 • **period duration:** 21 days • **daily prompt frequency:** 1 • **sampling type:** fixed signal-contingent (paper version was patient-initiative with possibility of daily reminder) • **prompt interval:** 1 day | none mentioned |
| Stone et al. (2016) | 7 | • pain intensity (adapted from PROMIS) • fatigue (adapted from PROMIS) • depression (adapted from PROMIS) • anxiety (adapted from PROMIS) | • **device (operating system):** patient-owned home computer • **application:** PROMIS Assessment Center | • **monitoring periods:** 1 • **period duration:** 28 days • **daily prompt frequency:** 1 • **sampling type:** interval-contingent with reminders available • **prompt interval:** not mentioned | none mentioned |
| Sztachańska et al. (2019) | • intervention group: 32 items • control group: 31 items | • daily affect (based on circumplex model) • daily self-esteem (based on Rosenberg Self-esteem sale) • daily acceptance of illness (Felton and Revenson, 1984) • daily optimism (Revised Life Orientation Test) • social support (Pasipanodya et al., 2012; Sarason et al., 1987) • coping (Endler and Parker, 1990) • gratitude (based on scale proposed by McCullough et al., 2002) | • **device (operating system):** not mentioned  • **application:** 63% provided data online (as opposed to paper) | • **monitoring periods:** 1 • **period duration:** 14 days • **daily prompt frequency:** 1 • **sampling type:** interval-contingent (in evening or early morning) • **prompt interval:** no prompt used | none mentioned |
| van den Berg et al. (2022) | 5 | ***5 item micro-surveys from RAND Short Form-36 (different items everyday):***  • physical functioning  • role limitations (physical and emotional health) • vitality (energy/fatigue) • emotional well-being • social functioning • pain • general health | • **device (operating system):** patient-owned smartphone (Android or iOS) • **application:** Beiwe | • **monitoring periods:** 1 • **period duration:** 224 days • **daily prompt frequency:** 1 • **sampling type:** not mentioned • **prompt interval:** not mentioned | none mentioned |
| van Roozendaal et al. (2023) | 20 | • fatigue (EnergyInSight app questionnaire)  • perpetuating coping factors (EnergyInSight app questionnaire)  • protective coping factors (EnergyInSight app questionnaire)  • other constructs not included in the article (EnergyInSight app questionnaire): positive and negative affect, social context, and physical activity | • **device (operating system):** patient-owned smartphone • **application:** EnergyInSight | • **monitoring periods:** 1 • **period duration:** 21 (with extensions if not at 75 completed assessments) • **daily prompt frequency:** 5 • **sampling type:** time-blocked random signal-contingent • **prompt interval:** not mentioned | none mentioned |
| Weaver et al. (2014) | not specified, presumably 8 | • temperature (adapted from CTCAE grades) • diarrhea (adapted from CTCAE grades) • vomiting and nausea (adapted from CTCAE grades) • mucositis (adapted from CTCAE grades) • hand-foot syndrome (adapted from CTCAE grades) • peripheral neuropathy (for other subgroup based on cancer type; adapted from CTCAE grades) | • **device (operating system):** Sony Ericsson c510 or Nokia 6303c mobile phones • **application:** software developed by University of Oxford and mobile phones programmed by OBS Medical, UK (also supplied server) | • **monitoring periods:** 8 • **period duration:** 21 • **daily prompt frequency:** 2 • **sampling type:** interval-contingent (in the morning and evening) • **prompt interval:** no prompt used | • **automated self-care advice** • **clinician alert:** nurse reviewed prioritised patient data and called to advise on care |
| Xu, Wang & Schoebi (2019) | not specified, at least 15 | • fear of cancer recurrence (adapted from Thewes et al., 2012) • daily couple communication (disclosure and perceptions of positive/negative information; based on Intimacy Process Model and Belcher et al., 2011) | • **device (operating system):** patient-owned smartphone • **application:** Wechat-message directed to browser-based survey | • **monitoring periods:** 1 • **period duration:** 10 • **daily prompt frequency:** 3 • **sampling type:** fixed signal-contingent • **prompt interval:** at least 3 hours | • **insight into responses**: after data completion, couples received a brief report of their fear of cancer recurrence and couple communication |
| Yap et al. (2013) | 2 | • vomiting frequencies (Shih et al., 2009; Yap et al., 2012) • nausea severity (CTCAE grades) | • **device (operating system):** patient-owned mobile phone • **application:** SMS (clinical SMS algorithm predeveloped in consultation with clinical pharmacists) | • **monitoring periods:** 1 • **period duration:** 5 • **daily prompt frequency:** 1 • **sampling type:** fixed signal-contingent • **prompt interval:** 1 day | • **automated self-care advice:** according to responses given  • **clinician alert:** in case of moderate or severe nausea or uncontrolled vomiting, an SMS was sent to pharmacist |

*Abbreviations:* BPI = Brief Pain Inventory; PANAS-X = Positive and Negative Affect Schedule – Expanded Version; SMS = Short Messaging Service; EORTC QLQ-C15-Pal = European Organisation for Research and Treatment of Cancer Quality of Life Questionnaire – Core 15 – Palliative version; CTCAE = Common Terminology Criteria for Adverse Events; PROMIS = Patient-Reported Outcomes Measurement Information System; FACT-L = Functional Assessment of Cancer Therapy – Lung version + IES-6 = Impact of Event Scale + BESI-C = Behavioral and Environmental Sensing and Intervention for Cancer
