# Supplementary material for: Intensive Longitudinal Methods Among Adults With Breast or Lung Cancer: Scoping Review
Source: J Med Internet Res. 2024 Jun 12;26:e50224. doi: 10.2196/50224 (PMC11208836; doi:10.2196/50224)
Supplement: Multimedia Appendix 4 [file jmir_v26i1e50224_app4.docx]

**Multimedia Appendix 4.** Response-related characteristics

| **Study** | **Participation rate**† | **Attrition**‡ | **Compliance**§ | **Monetary incentives** |
| --- | --- | --- | --- | --- |
| Aigner et al. (2016) | 34.18% | • **other attrition indicators:** 10% did not complete any diaries | • **compliance:** 73% | not mentioned |
| Badr et al. (2010);  Badr et al. (2013);  Stephenson et al. (2019) (reporting on the same study) | 62.80% | • **other attrition indicators:** 24% of couples lost data due to not charging battery | • **compliance:** 69.78% (*SD* = 21.93) | up to $80 each, based on percentage of completed assessments |
| Belcher et al. (2011); Pasipanodya et al. (2012) (reporting on the same study) | 41.80% | • **other attrition indicators:** 83.3% couples had enough data for inclusion in analyses (at least 4 diaries) | **• Belcher et al. (2011):**   • ***other compliance indicators:*** 74% patients completed all surveys; 83.3% couples completed at least 4 surveys **• Pasipanodya et al. (2012):**  • ***other compliance indicators:*** average number of completed assessments per patient: 6.98 and partner: 6.67 (some participants completed multiple assessments per day) | up to $130 per couple ($25 baseline, $5 per diary, $5 bonus for all 7) |
| Otto et al. (2015) - combination of two datasets, including Belcher et al. (2011) | • **study 1:** 41.80%;  • **study 2:** not mentioned | • **study 1:** 83.3% couples had enough data (at least 4 diaries) • **study 2:** not mentioned | • **compliance:** 49.42% across both studies, including both patients and their partners | • **study 1:** up to $130 per couple ($25 baseline, $5 per diary, $5 bonus for all 7) • **study 2:** not mentioned |
| Besse et al. (2016) | 76.47% | • **attrition:** 30.77% | • **compliance:** 62% | not mentioned |
| Cai et al. (2020) | not mentioned | • **attrition:** 0% | • **compliance:** 83.67% | $50 for providing feedback after diary period |
| Carson et al. (2021) | 37.20% | • **other attrition indicators:** 76.2% filled in at least one diary | • **compliance:** 71%, calculated using participants with at least one diary | $40 |
| Chumbler et al. (2007) | 83% | • **attrition:** 37.04% | • **compliance:** 84% | not mentioned |
| Çınar et al. (2021) | not mentioned | not mentioned | not mentioned | not mentioned |
| Coolbrandt et al. (2022) | 23.87% | • **attrition:** 5.5% at T2 (6-8 weeks after start treatment) | • **compliance:** 68.8% (at 3 weeks), 62.3% (6 weeks), 59.1% (12 weeks) | not mentioned |
| Dasch et al. (2010) | 33.00% | • **other attrition indicators:** 11.7% removed for extensive missing data or questionable study compliance | • **compliance:** 97.14%, calculated after removal of participants with extensive missing data or questionable study compliance | not mentioned |
| Dunsmore and Neupert (2023) | not mentioned | not mentioned | • **compliance:** 73% | online gift card (value unknown) |
| Hachizuka et al. (2010) | not mentioned | • **attrition:** 0% | • **compliance:** 90.3% (*SD* = 10.5); 80.2% for rescue medication beeps (*SD* = 16.8) | not mentioned |
| Harper et al. (2012) | 81.00% | none: variable study length | • **compliance:** 88% | not mentioned |
| Kearney et al. (2006) | not mentioned | • **other attrition indicators:** 16.67% did not complete pre-evaluation, 38.88% did not complete post-evaluation | not mentioned | not mentioned |
| Kim et al. (2016) | not mentioned | • **other attrition indicators:** 8.24% removed due to no biweekly completed assessments | • **compliance:** 44.20% for 24 weeks | not mentioned |
| Langer et al. (2018) | 50.70% | • **attrition:** 4.7% | • **compliance:** 88.8% | $75 check or gift card if ≥ 85% assessments completed, else $3 per completed beep |
| LeBaron et al. (2022);  LeBaron et al. (2023) (reporting on the same study) | not mentioned | • **attrition:** 42.9% | • **compliance:** not mentioned  • **other compliance indicators:** 283 user-initiated pain events (198 patient, 85 caregiver) | $50 gift card per dyad |
| Lee et al. (2023) | not mentioned | • **other attrition indicators:** 4% did not contribute data for any analysis | • **other compliance indicators:** 75% on day 1 to 42% on day 28 | not mentioned |
| Lim et al. (2022) | not mentioned | • **attrition:** 56.9% | • **compliance:** 95.7% | not mentioned |
| Maguire et al. (2005) | not mentioned | not mentioned | not mentioned | not mentioned |
| Maguire et al. (2015) | 28.10% | • **attrition:** 31.25% | not mentioned | not mentioned |
| McCall et al. (2008) | not mentioned | • **attrition:** 38.1% | not mentioned | not mentioned |
| McCann et al. (2009);  Kearney et al. (2009) (reporting on the same study) | not mentioned; recruited sample was 75% of target sample size | • **other attrition indicators:** 48.21% were not analysed at end of cycle 4 in both intervention and control group, 35.71% did not complete post-study questionnaires | not mentioned | not mentioned |
| Min et al. (2014) | not mentioned; 56.71% of recruited people signed informed consent | • **attrition:** 21.05% | • **compliance:** 45% | none |
| Mooney et al. (2014) | 85% | • **attrition:** 25.6% (22.5% treatment group, 28.9% control group) | • **compliance:** 65% for 223 participants | not mentioned |
| Nordhausen et al. (2022) | not mentioned | not mentioned | • **compliance:** 84.9% | not mentioned |
| Passardi et al. (2022) | not mentioned | • **other attrition indicators:** 12.5% were not evaluable for toxicity | • **other compliance indicators:** 33 once per week, median accesses per day was 2 (IQR: 1-3) | not mentioned |
| Pinto et al. (2021) | 60.53% | • **other attrition indicators:** 13% did not complete all assessments | • **compliance:** 78.62% | $30 + $1 for each assessment completion |
| Ratcliff et al. (2014) | not mentioned | • **attrition:** 0% • **other attrition indicators:** 4.76% did not complete any wake-time assessments | • **compliance:** 57% | based on percentage of complete EMAs (no further details mentioned) |
| Schuler et al. (2023) | 60% | • **attrition:** 0% | • **compliance:** morning questionnaire = 57%, smartwatch wear-time adherence = 73% | not mentioned |
| Shiyko et al. (2014);  Shiyko et al. (2019) (reporting on the same study) | • **Shiyko et al. (2014):** not mentioned • **Shiyko et al. (2019):** 81% | • **Shiyko et al. (2014):** • ***other attrition indicators:*** 11 dropped out (not indicated if 59 is remaining number or not) • **Shiyko et al. (2019):** • ***other attrition indicators:*** analyzable data from 59/71 (none quit the study) | • **Shiyko et al. (2014):**   • ***other compliance indicators:*** 81.4% of participants had 100% compliance, remaining had on average 65% (1544 assessments on 59 = 93.46%) • **Shiyko et al. (2019):**   • ***compliance:*** 61% (SD = 25) | not mentioned |
| Solk et al. (2019);  Phillips et al. (2020);  Auster-Gussman et al. (2021);  Welch et al. (2023); Whitaker et al. (2023) (reporting on the same study) | 23.58%; 17.33% of participants who consented did not complete all periods | • **other attrition indicators:** 84% completed all time points; 67 of 75 completed at least one | • **compliance:** 84.14% | $100 at each time point, regardless of compliance |
| Steffen et al. (2018);  Steffen et al. (2020) (reporting on same study) | 90.30% | • **other attrition indicators:** 89.3% of consented provided baseline and ≥ 7 diaries | • **compliance:** 98% | $30 initial questionnaire, $3 each diary, $4 each completed week, $6 all 21 days completed (in the form of gift cards) |
| Stone et al. (2016) | not mentioned | • **other attrition indicators:** 1.2% dropped out or had incomplete data | • **compliance:** 93.92% | $200 study completion, $150 lottery (for every 25th person in study) to win when person completed every assessment |
| Sztachańska et al. (2019) | not mentioned | • **other attrition indicators:** 9.84% changed mind before daily assessments, 21.31% provided less then 5 days of data | • **compliance:** 95.92%, calculated after removing people with less than 5 completed assessments | not mentioned |
| van den Berg et al. (2022) | not mentioned | • **other attrition indicators:** 45% did not complete surveys preoperatively, 22% postoperatively (0-6 weeks), 67% late postoperatively (after 6 weeks) | • **compliance :** 58.48% | none |
| van Roozendaal et al. (2023) | 25.87% | • **attrition:** 18.91% | • **other compliance indicators:** between 68 to 101 completed assessments per person (M = 87.7; SD = 10.0) | not mentioned |
| Weaver et al. (2014) | 66.67% | • **other attrition indicators:** 23.08% completed 8 cycles of treatment | • **compliance:** 92.6% | not mentioned |
| Xu, Wang & Schoebi (2019) | 27.65% | • **attrition:** 10% | • **compliance:** 75.9% | not mentioned |
| Yap et al. (2013) | 37.60% | • **attrition:** 11.8% | • **other compliance indicators:** 73.3% completed all 5 assessments. compliance on day 1: 78.3% and compliance on day 5: 83.3% | not mentioned |

† calculated as ‘number of informed consents signed’ / ’number of participants asked to take part in study’. Other participation indicators mentioned if needed data unavailable for calculation.

‡ calculated as 1 – (‘number of participant who completed the full study period’ / ‘the number of informed consents signed’). Other attrition indicators mentioned if needed data unavailable for calculation.

§ calculated as ‘total number of completed assessments’ / ‘total number of scheduled assessments’. Other compliance indicators mentioned if needed data unavailable for calculation.
